# Engineering of Small Ribozymes Acting on RNA: What is Needed to Make a New Function Work with an Existing Catalyst?

**DOI:** 10.1002/cbic.202500213

**Published:** 2025-05-21

**Authors:** Constanze Ebermann, Sabine Müller

**Affiliations:** ^1^ Institute of Biochemistry University of Greifswald Felix‐Hausdorff‐Str. 4 17489 Greifswald Germany

**Keywords:** engineering, hairpin ribozymes, rational designs, ribozymes, RNA

## Abstract

The engineering of nucleic acids has been a longstanding objective in research, with the field gaining significant attention following the discovery of ribozymes in the early 1980s. Numerous nucleic acid catalysts have been developed to catalyze a wide range of reactions, and the structures of ribozymes have been modified to allow allosteric regulation by an external cofactor. All these constructs hold considerable promise for applications in biosensors for medical and environmental diagnostics, as well as in molecular tools for regulating cellular processes. In addition to the development of nucleic acid enzymes through in vitro selection, rational design offers a robust strategy for engineering ribozymes with customized properties. The structures and mechanisms of numerous nucleic acid catalysts have been thoroughly elucidated, making structural modulation a viable approach for designing their functional properties. Rational design necessitates the consideration of several parameters, and a range of tools is available to guide sequence design. This review discusses sequence, structural, and functional design, primarily using the example of the hairpin ribozyme, to highlight the challenges and opportunities of rational nucleic acid enzyme engineering.

## Graphical abstract

Rational design is a potent strategy for developing ribozymes with advanced functionalities. Engineered variants of the hairpin ribozyme impressively demonstrate how function can be tuned through structural manipulation.

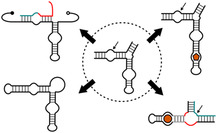

## 1 Introduction

Over the past three decades, the development of nucleic acid enzymes has emerged as a significant domain of research, with potential applications in the fields of chemical and molecular biology, as well as medical and environmental diagnostics.^[^ ^1^, ^2^ ^]^ Nucleic acid catalysts, ribozymes and DNAzymes, are versatile molecular tools, and their importance for the aforementioned research domains has steadily increased in recent years. Ribozyme applications in molecular biology range from simple cleavage or ligation of a defined RNA target to the introduction of sequence changes and/or modifications in the desired target RNA and the regulation of gene expression in combination with a suitable sensor module (e.g., an aptamer).^[^ ^3^, ^4^, ^5^, ^6^ ^]^ Two main strategies are employed for nucleic acid enzyme engineering: The first of these is in vitro evolution, which is based on the selection of a nucleic acid molecule with the desired properties from a library of random sequences. The second is rational design, which starts from a known ribozyme and is based on structural manipulation to affect the function in a predefined way. The former approach has facilitated the development of numerous nucleic acid enzymes with novel activities, thereby expanding the repertoire of nucleic acid catalysis.^[^ ^7^, ^8^, ^9^ ^]^ In contrast, the latter has focused more on exploiting the intrinsic catalytic properties of ribozymes for novel developments, with a thorough understanding of structure and mechanism being of utmost importance. A substantial body of research has been dedicated to the collection of data on the structure and mechanism of nucleic acid enzymes.^[^ ^4^, ^10^, ^11^ ^]^ This has led to a comprehensive understanding of RNA‐ and DNA‐based catalysts, which have been transformed into practical tools. Thus, in recent years, significant advancements have been made based on the utilization of established nucleic acid catalytic structures.^[^ ^4^, ^12^ ^]^ For instance, ribozymes have been engineered to facilitate RNA circularization^[^ ^13^, ^14^ ^]^ and several ribozymes and DNAzymes have been designed to be regulated by allosteric cofactors or temperature, and as such find application as tools in environmental and medical diagnostics or even therapies.^[^ ^1^, ^2^, ^5^, ^12^, ^15^, ^16^ ^]^

In our laboratory, over the past years, we have developed hairpin ribozyme derivatives that support RNA processing, such as recombination and splicing, repair, oligomerization, and circularization.^[^ ^17^, ^18^, ^19^, ^20^, ^21^, ^22^, ^23^, ^24^ ^]^ Looking at such developed nucleic acid catalysts, designed by us and others, it is impressing to see, how well they perform in fulfilling their intended function. However, it is noteworthy that achieving this level of performance typically necessitates a substantial investment of time and effort. Even when adapting a well‐characterized ribozyme for a novel application, numerous hurdles and challenges persist, including sequence design, site specificity, structure design, and target accessibility. Consequently, there is a clear need for comprehensive guidelines to facilitate the design of novel ribozyme‐based applications, ensuring the attainment of a functional system. The design process can be categorized into three primary components. The initial component pertains to the sequence design, the second addresses structural considerations necessary for a specific application, and the third component, functional design, is instrumental in the identification of novel activities.

In this review article, the focus will be on naturally occurring small self‐cleaving ribozymes as the starting point for the design. In particular, the engineering of hairpin ribozyme variants will be discussed, as the hairpin ribozyme is a well‐studied small catalytic RNA that has been used in our laboratory for a number of engineering projects.

## 2 Small Self‐Cleaving Ribozymes

### 2.1 General Mechanism

Since the discovery of the hammerhead ribozyme as the first small self‐cleaving ribozyme in 1986,^[^ ^25^ ^]^ a plethora of other ribozymes with self‐cleaving activity have been identified and described. These ribozymes are predominantly found in or associated with viral RNA, or in highly conserved and rather ancient regions of bacterial and eukaryotic, even human RNA, where they serve as functional elements in RNA self‐processing.^[^ ^4^ ^]^

**Figure 1:**
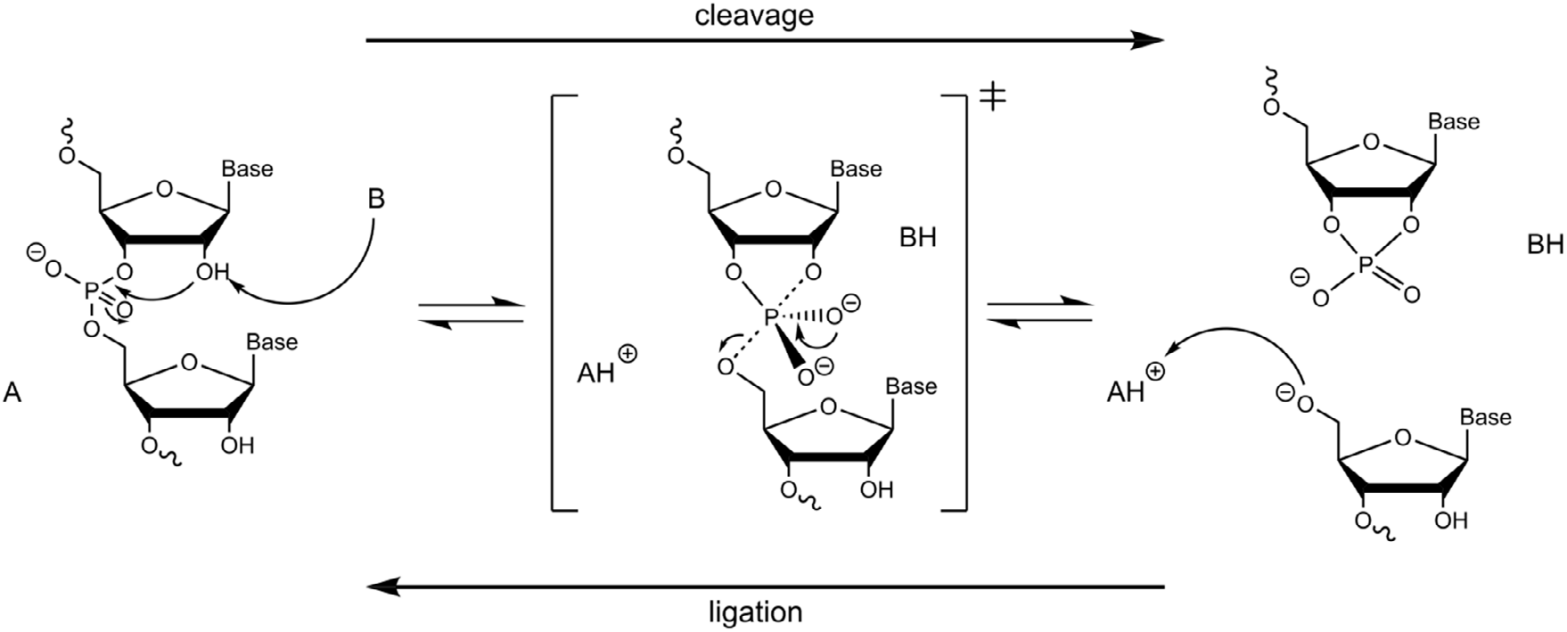
General mechanism of small self‐cleaving ribozymes, according to the principle of general acid‐base catalysis. General acid (A) and base (B).

Despite the considerable variation in the structures and catalytic strategies of the various small self‐cleaving ribozymes that have been characterized to date, the underlying mechanism remains the same. In their natural environment, these ribozymes facilitate the reversible cleavage of an RNA strand in *cis* (i.e., the same RNA strand). However, they can also be engineered to act as true catalysts to cleave or ligate a substrate strand in *trans*. Either way, the ribozymes facilitate a transesterification reaction by general acid–base catalysis (**Figure** **1**), with either a nucleobase within the ribozyme structure, hydrated magnesium ions, or external cofactors playing the role of general acid or base.^[^ ^4^, ^26^ ^]^ The general base deprotonates the 2′‐OH group, thereby activating the oxygen for nucleophilic attack on the adjacent phosphorus, resulting in the formation of a trigonal bipyramidal intermediate. The 5′‐OH is then protonated by a general acid, resulting in a good leaving group. Upon cleavage, the typical products are released, one fragment with a free 5′‐OH group and the other with a 2′,3′‐cyclic phosphate. Ligation proceeds along the same pathway in the opposite direction, resulting in the rejoining of two fragments with the aforementioned end functionalities (5'‐OH and 2′, 3′‐cyclic phosphate).^[^ ^27^ ^]^

The majority of these ribozymes are dependent on magnesium ions for optimal functionality. The primary function of Mg^2+^ is to facilitate the appropriate folding of the ribozyme into its active tertiary structure by counteracting the negative charge of the phosphate backbone.^[^ ^28^, ^29^, ^30^ ^]^ However, as mentioned above, the hydrated magnesium ions can also act as a general acid or base, directly contributing to the catalytic mechanism, or as a Lewis acid, interacting with specific residues in the catalytic core and thus stabilizing products or intermediates of the reaction.^[^ ^28^ ^]^

### 2.2 The Hairpin Ribozyme

The hairpin ribozyme (HPR) was discovered in the negative strand of tobacco ringspot virus satellite RNA, where it plays a critical role in processing intermediates of rolling circle replication.^[^ ^31^ ^]^ Since then, the HPR has been the subject of extensive research, leading to the development of various modified versions for optimized performance in a range of applications. The crystal structures of several variants have been solved,^[^ ^32^, ^33^ ^]^ and the sequence and structural requirements for proper catalytic performance have been thoroughly studied.^[^ ^34^ ^]^

**Figure 2:**
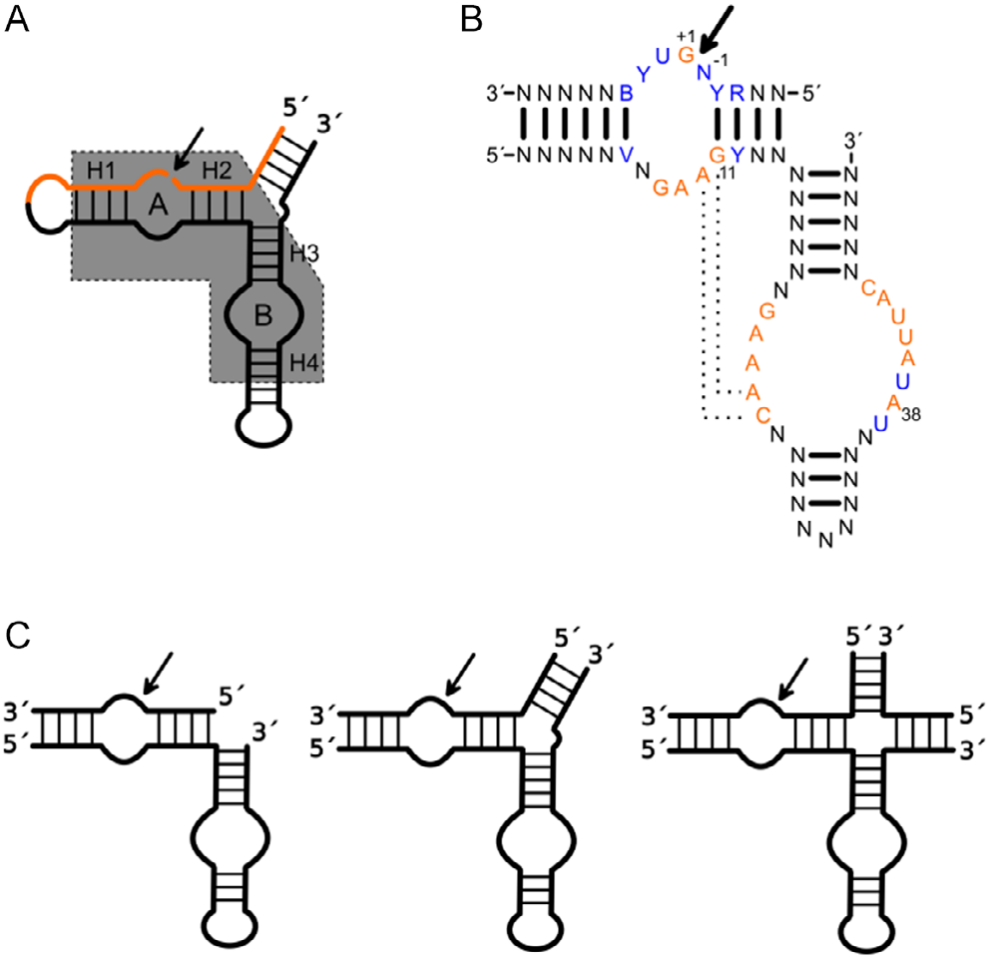
Structural variants of the hairpin ribozyme. A) General structure of a TWJ hairpin ribozyme with loops A and B and helices (H) 1–4. Minimal motif highlighted in gray. The substrate strand is indicated in orange, and the HPR strand in black. The cleavage site is indicated by a black arrow. B) Consensus sequence of the minimal HPR. Orange: highly conserved nucleotides; blue: semi‐conserved nucleotides. The ribose zipper is indicated by dashed lines, the cleavage site is indicated by a black arrow. C) The HPR as minimal motif (two‐way junction), TWJ, and FWJ (left to right).

**Figure 3:**
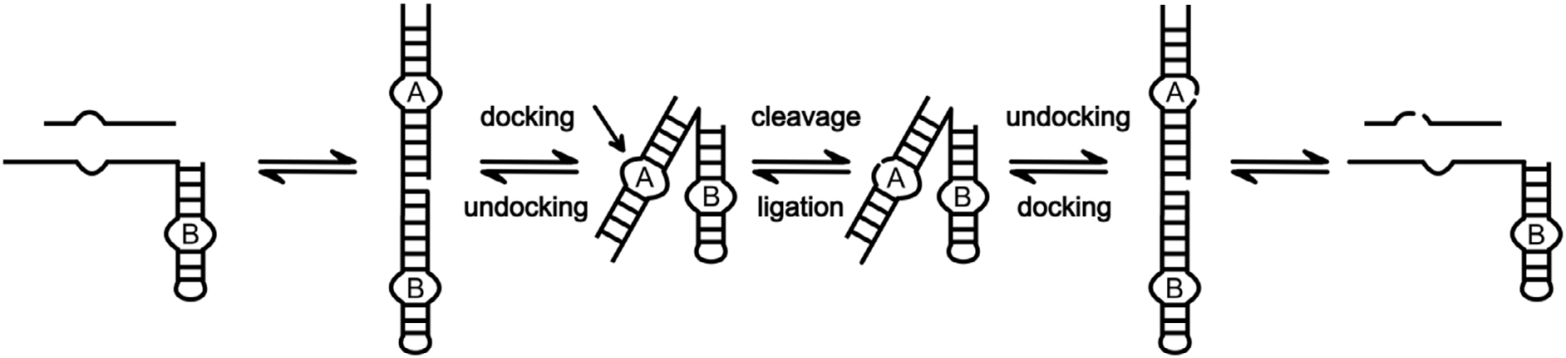
Conformational changes of the HPR during the reaction cycle. Note, substrate/product binding and release can occur only from the extended state, while reaction proceeds only from the docked state.

The wild‐type HPR forms a four‐way junction (FWJ) with four partially double‐stranded arms.^[^ ^35^ ^]^ However, the minimally functional HPR consists of only two of these arms connected by a hinge region, resulting in a two‐way junction structure. The two arms each consist of two helical regions interrupted by a loop (**Figure** **2**A). The cleavage site is located in loop A between the guanosine at position +1 (G^+1^) and the upstream base (B^−1^) within the consensus sequence N*GUC.^[^ ^36^ ^]^ For the rational design of the HPR, some sequence constraints must be taken into account.^[^ ^37^, ^38^ ^]^ While the sequence of the helical regions exhibits significant variability, nucleobases in loops A and B are strongly conserved (Figure 2B). It is important to note that for proper folding and activity, helix 1 should span a minimum of four base pairs, while helix 2 is required to have a precise length of four base pairs.^[^ ^39^ ^]^ The high conservation of the two loops is due to their important role in folding the HPR into its catalytically active structure and formation of the catalytic core. The interaction of bases in the loops, particularly through a ribose zipper and an interdomain Watson–Crick base pair, results in the close proximity of the loops, a process referred to as docking.^[^ ^40^ ^]^ This leads to a bent conformation of the ribozyme (**Figure** **3**).^[^ ^41^ ^]^ Cleavage and ligation of the HPR occurs from the docked state, while binding/dissociation of the substrates/products is only possible from an extended state, requiring the ribozyme to dynamically transit between the docked and extended conformation.^[^ ^42^ ^]^ Ligation requires a stable docked state, whereas cleavage can also occur from a transiently docked conformation. If the lifetime of the docked state is too short, dissociation of the substrates is likely to occur too rapidly for productive ligation. Therefore, ligation of two strands is enhanced by structural elements or environmental conditions that stabilize the docked state.^[^ ^43^ ^]^ The rates of hairpin ribozyme cleavage and ligation are strongly pH dependent, consistent with proton transfer occurring in the transition state.^[^ ^44^, ^45^ ^]^ Typically, observed cleavage rates are in the area of 0.1–1 min^−1^, dependent on the specific ribozyme structure and conditions, whereas corresponding ligation rates are about 10‐ to 30‐fold higher.^[^ ^39^, ^41^ ^]^ Strikingly, an internal equilibrium constant between cleavage and ligation *K*_int_ = *K*_lig_/*K*_cleav_ of 34 was determined, showing that reaction is substantially biased toward ligation.^[^ ^45^ ^]^

As mentioned above, the docked state is stabilized by tertiary interactions within the folded structure. Additional stabilization can result from the specific junction structure organizing two (minimal motif), three, or four arms (Figure 2C). In its natural context, the hairpin ribozyme is embedded into a FWJ, which assists folding in the docked structure with close contact between loops A and B. On the opposite, in the minimal two‐way junction HPR, the contribution of the junction (which is rather a hinge) is virtually zero. In between is a three‐way junction (TWJ) structure, which by the additional arm and a short bulge at the appropriate position helps fold and stabilize the docked state.^[^ ^46^, ^47^ ^]^ However, careful design of the sequence around the junction and of the bulge is of utmost importance to achieve the intended structural effects. This has been well considered in our designs. Like all small self‐cleaving ribozymes, the HPR requires magnesium ions for activity. However, for the HPR, magnesium ions are not integral to the catalytic mechanism per se. Here the metal ions function as a charge buffer, neutralizing the negative charges of the ribozyme phosphate backbone and enabling proper folding. Thus, functional HPR catalysis can occur in the absence of magnesium ions under various conditions.^[^ ^46^, ^48^, ^49^, ^50^ ^]^ As the strengths of docking increases with the number of arms involved in the junction, FWJ HPRs show higher activity and are active at lower magnesium ion concentrations than TWJ HPRs or the minimal two‐way junction motif.^[^ ^47^, ^51^ ^]^ As a consequence, FWJ HPRs tend to promote ligation, while the equilibrium shifts toward cleavage as the number of junction arms decreases (Figure 2C).

### 2.3 The Hammerhead Ribozyme

**Figure 4:**
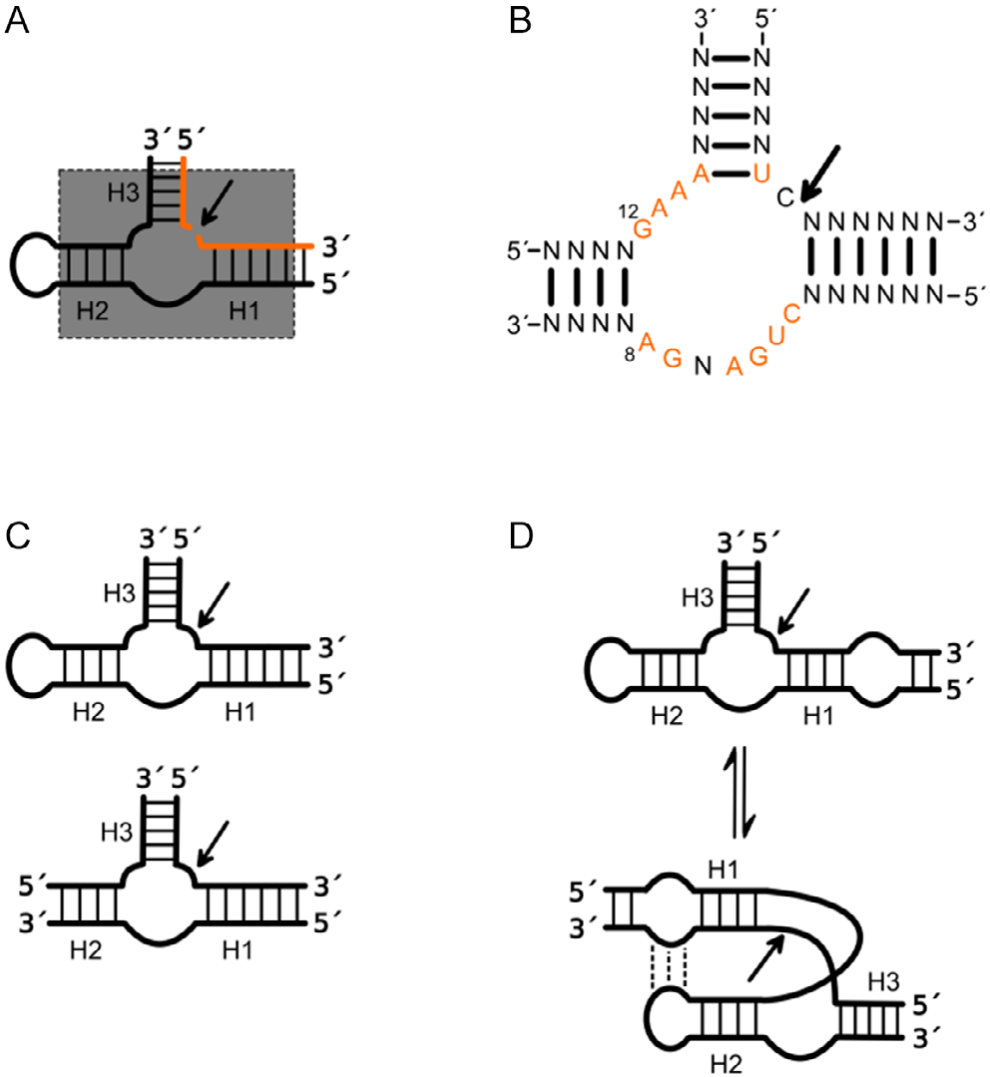
Structural variants of the hammerhead ribozyme (HHR). A) General structure of HHR with stems/helices (H) 1–3. The minimal motif is highlighted in gray. The substrate strand is indicated in orange, and the HPR strand in black. The cleavage site is indicated by a black arrow. B) Consensus sequence of the minimal HHR. Orange: highly conserved nucleotides. The cleavage site is indicated by a black arrow. C) HHR variants with open or closed stems. D) Structure of the wild‐type HHR with an additional loop in H1 and folding supported by loop–loop interaction. The cleavage site is indicated by a black arrow.

The hammerhead ribozyme (HHR) was the first small self‐cleaving ribozyme to be discovered, and as such, it has been the focus of significant research.^[^ ^52^ ^]^ It has been found in satellite RNAs of plant viruses and plant viroids.^[^ ^53^ ^]^ Like the HPR, it plays a functional role in the life cycle of these RNAs during rolling circle replication. The HHR structure has been determined by crystallography and consists of a junction region with three helices/stems, with helix 2 as an open or hairpin stem structure (**Figure** **4**).^[^ ^54^ ^]^ G8 and G12 in the catalytic strand are required for catalytic activity and are therefore highly conserved (Figure 4B), while the sequence of the helical regions is variable.^[^ ^55^ ^]^ The cleavage site is located in the junction region between stems I and III within the sequence UC*N. Tertiary interactions between stems I and II are crucial for optimal activity.^[^ ^56^ ^]^ These interactions are facilitated by a loop–loop interaction between stems I and II in the full‐length HHR (Figure 4D).^[^ ^57^ ^]^ However, the minimal motif lacks the loops in stem I and II, resulting in the absence of this interaction in the minimal structure. Nonetheless, as the tertiary contact is not essential for activity, the minimal HHR is still able to cleave its substrate, but has a much lower turnover rate and is also dependent on higher concentrations of magnesium ions than the wild type.^[^ ^58^, ^59^ ^]^

Ligation activity has been measured for a few hammerhead ribozymes, but in all cases, it was rather poor. The rate constant of ligation is on average 0.01 min^−1^, while the rate constant for cleavage is about 1 min^−1^.^[^ ^60^, ^61^, ^62^ ^]^ It has been proposed that the strong preference for the cleavage reaction is caused by the flexibility of the complex of the hammerhead ribozyme with two ligation substrates, which provides an entropic advantage for cleavage.^[^ ^60^ ^]^ The design of conformationally restricted, more rigid hammerhead ribozymes resulted in increased ligation activity,^[^ ^63^, ^64^, ^65^ ^]^ which however, was still not high enough to motivate the practical use of the hammerhead ribozyme for ligation. As a result, hammerhead ribozymes have been used primarily for RNA cleavage.

At the example of the hammerhead ribozyme, it has been demonstrated recently that the evolutionary trajectory of each nucleotide in the catalytic core directly correlates with their functional importance, potentially giving researchers a novel method to assess the sequence requirements of functional nucleic acids.^[^ ^66^ ^]^

### 2.4 Other Small Self‐Cleaving Ribozymes

Despite the fact that other small self‐cleaving ribozymes have received comparatively less consideration in the context of rational engineering, it is important to note that HPR or HHR may not be optimal for specific applications, thus necessitating the consideration of alternative catalysts. The overarching principles and guidelines for rational engineering outlined herein can be adapted to accommodate other small ribozymes, with the same aim of engineering a specific function into existing catalytic RNAs. This includes the Twister, Twister Sister, Pistol, Hatchet, Hepatitis Delta Virus (HDV), and Varkud Satellite (VS) ribozymes. The structures and mechanisms of these ribozymes have been extensively reviewed.^[^ ^4^ ^]^

For further information, we refer to the online platform Ribocentre (https://www.ribocentre.org/), where comprehensive information on all natural ribozymes can be found.

### 2.5 Manipulation of Functionality by Reaction Conditions.

Ribozyme activity is dependent on intrinsic properties, such as sequence and structure, as well as on external factors including the experimental setup and reaction conditions. Thus, the catalytic efficiency and the internal cleavage‐ligation equilibrium of the ribozyme are influenced by several factors, including the ratio of ribozyme to substrate(s), the temperature, the concentration and species of available metal ions, and the presence and amount of additives, that is, crowding agents. To explain how these parameters influence the activity of ribozymes we here focus on the hairpin ribozyme, as it is the sole small self‐cleaving ribozyme for which substantial ligation activity has been documented so far.

#### 2.5.1 Ribozyme–Substrate Ratio

The outcome of a ribozyme‐catalyzed reaction is dependent on the ratios of the ribozyme and the substrate species involved, not only in terms of differences in reaction kinetics, but also regarding the qualitative result of more complex reaction scenarios. For instance, in the context of a recombination reaction (see Chapter 4), utilizing an excess of one substrate over another would enhance the probability of this fragment competing successfully with the other for ribozyme binding, thereby promoting its cleavage or ligation. In a different scenario, the substrate(s) themselves can act as oligonucleotide effectors (see also Chapter 5.1) to induce different catalytic folds of an RNA. Depending on the substrate concentration and ratio as well as the substrate‐ribozyme ratio, the one or the other fold can be preferred, and with that also the preference for cleavage or ligation is determined.

#### 2.5.2 Temperature

Due to their natural origin, ribozymes exhibit optimal activity at 37 °C. However, they are also capable of functioning at slightly lower or higher temperatures. The primary consideration is that the catalytically competent conformation remains intact (requiring a temperature that is not excessively high), and the necessary conformational dynamics are maintained (requiring a temperature that is not excessively low). The temperature regime also influences the preference for cleavage or ligation. As mentioned previously, HPR ligation requires a stable docked conformation, whereas cleavage can occur from a transiently docked state.^[^ ^42^, ^43^ ^]^ Accordingly, ligation is favored at lower temperatures, while cleavage is preferred at higher temperatures. Hence, HPR cleavage reactions typically proceed at 37 °C, while ligation protocols often involve lowering the temperature to ≈20 °C,^[^ ^18^ ^]^ in some cases even below 0 °C.^[^ ^50^, ^67^, ^68^ ^]^

#### 2.5.3 Metal Ions

The availability of metal ions (typically magnesium) is pivotal for RNA folding and, consequently, catalytic activity. Thus, it is feasible to regulate ribozymes by modulating the concentration of these ions. Under appropriate conditions, ribozyme catalysis can occur in the absence of magnesium ions.^[^ ^46^, ^48^, ^49^, ^50^ ^]^ Studies on the dependence of ribozyme activity on metal ions revealed that Mg^2+^ can be substituted by alternative metal ions, including rather unconventional candidates such as transition metals, resulting in enhanced activity.^[^ ^69^ ^]^ It is important to consider that the in vivo environment typically exhibits a magnesium ion concentration that is approximately tenfold lower than the levels normally used in standard in vitro functionality assays. The physiological magnesium ion concentration is ≈1 mm, whereas ribozyme reactions in vitro are typically performed at around 10 mm or even higher concentrations, depending on the specific ribozyme. In addition, as previously stated, the minimal motifs of ribozymes exhibit a greater dependence on magnesium ions compared to their wild‐type counterparts within a more complex structural environment. The absence of stabilizing interactions or structural elements in minimal motifs can be, at least partially, compensated by elevated magnesium ion concentrations.

#### 2.5.4 Crowding Agents

It has been demonstrated that some compounds have the capacity to densify the environment of the ribozyme, thereby mimicking a cellular setup in which all reaction components are more closely packed than in a setup where free diffusion is possible.^[^ ^70^ ^]^ This effect is known as molecular crowding. Polymers with a high molecular weight, such as polyethylene glycol (PEG) or polypeptides, as well as smaller compounds, including amino acids and nucleotides, have been shown to facilitate this crowding effect. It has been demonstrated that molecular crowding can promote the correct folding of ribozymes, even under conditions that would otherwise lead to a decrease or complete inhibition of activity.^[^ ^43^ ^]^ Additionally, it has been observed that the poor ligation activity of ribozymes at low magnesium ion concentrations or under denaturing conditions can be restored by molecular crowding agents, including amino acids and nucleotides.^[^ ^71^ ^]^

## 3 Rational Engineering of Ribozymes

### 3.1 General Aspects/Rules

In the field of ribozyme engineering, two general approaches are commonly employed. The forward approach finds application when a specific partition of a target sequence to be modified is known. Conversely, when a sequence is required that assumes a particular secondary structure, yet there are no specific sequence restrictions, the inverse approach is utilized. However, in practice, a combination of both approaches is often employed due to the presence of sequence patches that are restricted to specific nucleotides and components of random sequence. In the process of designing a ribozyme with a specific application in mind, two fundamental aspects must be taken into consideration: the sequence and the structure. Given the interdependence of RNA secondary and tertiary structure on the length and sequence of the constituent strands, these two aspects cannot be examined independently. To ensure a successful design, it is crucial to carefully examine and investigate base‐pairing, non‐canonical interactions, tertiary contacts, and alternative folds or binding potentials. When designing a ribozyme, it's crucial to keep the catalytic center intact. Accordingly, the substrate strands must contain the highly conserved nucleotides that are essential for catalysis or maintaining the active fold. For the HPR, the substrate sequence requirements are N*GUC, and for the HHR, XC*N at the cleavage site. Furthermore, nucleotides involved in specific tertiary interactions and in the formation of the catalytic center need to be preserved (Figure 2 and 4). Changes to the sequence in the helical regions are more likely to be tolerated without disturbing the activity, as long as base pairing is retained.^[^ ^22^, ^72^ ^]^

The minimal sequence and structure of the ribozyme are the starting point for modification to achieve the desired functionality. After careful engineering of the core sequence, structural parameters are taken into account. For the HPR, the equilibrium between cleavage and ligation can be adjusted by changing the lengths of the substrate binding arms and thus of the helix lengths, with longer helices being advantageous for ligation. On the opposite, the insertion of mismatches or bulges has been shown to result in the destabilization of substrate/product binding, thereby favoring cleavage.^[^ ^17^, ^73^, ^74^ ^]^ It is evident that longer helical regions facilitate enhanced substrate specificity in terms of recognizing a desired target sequence. Consequently, the trade‐off between specificity and diffusion likelihood emerges as a critical consideration, particularly when multiple turnover is desired. This principle finds application in the context of ribozyme‐mediated recombination of two substrate strands, as discussed in Chapter 4. If the binding of the recombination product to the ribozyme is too strong, it cannot be replaced by a new substrate strand, and the ribozyme can perform only one recombination reaction. Additionally, if the recombination product remains bound to the still‐active ribozyme, it is even possible that it is cleaved again.

The selection of a particular junction structure, such as a minimal motif, a TWJ, or an FWJ, has been demonstrated to influence the cleavage or ligation preference.^[^ ^75^ ^]^ This is attributed to the nature of the junction affecting the stability of the docked complex and, consequently, the ligation activity of the HPR. We have used these considerations in the past in a number of HPR designs.^[^ ^6^, ^19^, ^20^, ^21^, ^76^ ^]^ A study that combined computational modeling and FRET (Förster resonance energy transfer) experiments to investigate the junction region of the HPR revealed how the topology of that region affects the folding stability of the ribozyme. The study showed that adding a short, single‐stranded linker between helices 3 and 5 of a TWJ‐HPR could improve how efficient it is at docking and, as a result, how active the ribozyme is. The results also show the benefits of using models to design ribozymes, in this case the TOPRNA software for predicting junction topologies.^[^ ^77^ ^]^

Subsequent to the completion of the initial draft of the sequence of ribozyme and substrate(s), a secondary structure check should be conducted to ascertain the correct folding of the substrate‐ribozyme complex and the precise binding location and orientation of the substrates. To facilitate this process, several algorithms are available (see below) that can predict RNA secondary structure. It is important to acknowledge that the outputs of these algorithms are probabilities, and as such, the results are not entirely accurate. However, they can serve as a valuable guideline for the design of the sequence.

### 3.2 Software Aided Design

As mentioned earlier, there are two different approaches to engineering RNA sequences (in our work particularly ribozymes). The forward approach uses a known sequence to predict the structure. The inverse approach starts from the desired secondary structure to find a sequence that fits that structure. There are now a large number of algorithms available for both approaches (**Table** **1**).

**Table 1:** Comparison of forward and inverse approach algorithms for RNA structure prediction.

	Name	Web application/server	Software installation required (X) or optional (O)
Forward approach	RNAfold	X	–
	RNAstructure	X	O
	Mfold/UNAFold	X	–
	NUPACK	X	–
	TOPRNA	–	X
Inverse approach	RNAifold	X	–
	MODENA	–	X
	NUPACK	X	–
	antaRNA	X	–
	RNAinverse	X	–
	Enzymer	–	X
	RNA Designer	X	–
	GHOST‐NOT/GHOST‐YES	–	X
	Ribosoft	X	–

For ribozyme design, the forward approach is usually more useful, because of the conserved parts of the sequence that need to be precisely defined. However, the inverse approach is also applicable for a first sequence design for highly variable regions.

Once a first sequence design is in place, structure prediction algorithms (forward approach) can be used to check that the folding of the whole sequence (ribozyme and substrate together) is as desired. If the predicted structure deviates from the correct fold, mutations can be introduced or the length of helical regions can be changed. In this way, after each change is made, the structure should be predicted again. This should be done iteratively until the sequence leads to a predicted structure that matches the desired fold. In all these steps, it is also important to ensure that the desired substrate binds to the ribozyme in the correct position and orientation. The ribozyme and substrate strands should also be examined independently to check for alternative undesired interactions, such as self‐duplexing of the ribozyme or substrate strands.

A number of different algorithms are available for both forward and inverse approach, and new ones are being developed and refined. It is often possible to access these applications directly from a web server without having to install them on a computer. A selection of commonly used programs is listed in Table 1.

For example, we used the NUPACK software to design a self‐splicing hairpin ribozyme (see Section 4.2).^[^ ^20^ ^]^ Specifically, the NUPACK design was used in an inverse approach to calculate oligonucleotide sequences that are forced to fold into more than one defined secondary structure. We created a short script to compute a set of RNA sequences that fit our defined rules for a self‐splicing hairpin ribozyme. The best variant performed as well as a corresponding hairpin ribozyme variant designed by hand, although the design process using NUPACK was much faster and more straightforward.

Another example of the successful application of the inverse approach to ribozyme design was recently published by Perreault et al.^[^ ^78^ ^]^ Using the inverse folding program “Enzymer,” the authors were able to generate functional hammerhead and *glmS* ribozymes containing a pseudoknot. This pseudoknot was desired to stabilize ribozyme folding and thus increase the activity. The conserved parts of the sequence were set as invariable seed regions, while all other nucleotides were left variable. The proposed pseudoknot ribozymes generated by the program were then experimentally investigated, and most of them were found to be active. Although the activity was not as high as for other published ribozymes, this study still serves as a proof of principle for the use of inverse folding algorithms in ribozyme design.

In a separate study, Penchovsky and coworkers employed algorithms to engineer ribozymes that can be activated or deactivated by the binding of an external oligonucleotide (for ribozymes with external effectors, see Chapter 5).^[^ ^79^ ^]^ To this end, the authors developed two programs, GHOST‐YES and GHOST‐NOT (Generator for High‐speed Oligonucleotide Sensing Allosteric Ribozymes). These programs utilize the extended version of the hammerhead ribozyme to generate ribozymes capable of being switched on or off, according to the user's preference, through the binding of an external oligonucleotide. To achieve this, a randomized oligonucleotide binding site (OBS) was incorporated into the HHR sequence. The GHOST‐NOT algorithm facilitates the generation of HHRs that fold into the OFF‐state upon oligonucleotide binding to the OBS within the ribozyme. Conversely, the GHOST‐YES algorithm generates ribozymes that fold into the active conformation (ON‐state) in the presence of the oligonucleotide. The two programs are capable of generating a set number of HHRs with varying OBS sequences and predicting the structures of the ribozymes in the presence and absence of their oligonucleotide effector. In contrast to the in vitro selection of ribozymes for a specific application, the in silico approach using the GHOST algorithms for generating customized ribozymes offers several advantages, including its speed, ease, and reduced labor intensity. Experimental investigation has revealed that the predicted ribozymes exhibit high cleavage rates in response to the presence or absence of their respective effector molecule, suggesting the potential application of the GHOST algorithms for the design of high‐speed allosteric ribozymes for diagnostic or therapeutic purposes.^[^ ^79^ ^]^

In addition to the predicted secondary structure of ribozymes, examining the tertiary structure is also useful for further investigation of correct folding. The webserver RNAComposer can generate a 3D model of a ribozyme based on the calculated secondary structure.^[^ ^80^ ^]^ This model can be aligned and compared to the crystal structures of characterized ribozymes deposited in the Protein Data Bank. It is important to note that all of the aforementioned computer‐aided approaches to rational design are predictions and will never be one hundred percent exact. In silico modeling of algorithms is inherently limited in its capacity to mimic the complexity of in vitro and in vivo systems, because it cannot account for the numerous parameters that are prevalent in biological systems and that influence RNA structure. Consequently, while computational predictions can serve as a guide during the design process, their validity must be substantiated through experimental validation.

RNA secondary structure prediction remains a challenging endeavor, and over the past decade, modern tools implementing deep learning techniques for RNA structure prediction have led to tremendous progress in this field, resulting in significant improvements in prediction accuracy. Although deep learning‐based approaches offer numerous advantages over traditional prediction approaches, strong efforts are still needed to overcome the challenges and inherent limitations of deep learning techniques.^[^ ^81^, ^82^ ^]^

## 4 Engineered HPR Variants for Splicing and Recombination.

### 4.1 Recombination

It is evident that the two fundamental reactions of small self‐cleaving ribozymes—cleavage and ligation—can be leveraged to engineer these ribozymes for expanded functionalities. With our interest in mimicking RNA‐supported reactions in early life (RNA world), we were particularly interested in ribozyme design for RNA recombination and splicing.^[^ ^18^, ^19^, ^20^, ^21^ ^]^

**Figure 5:**
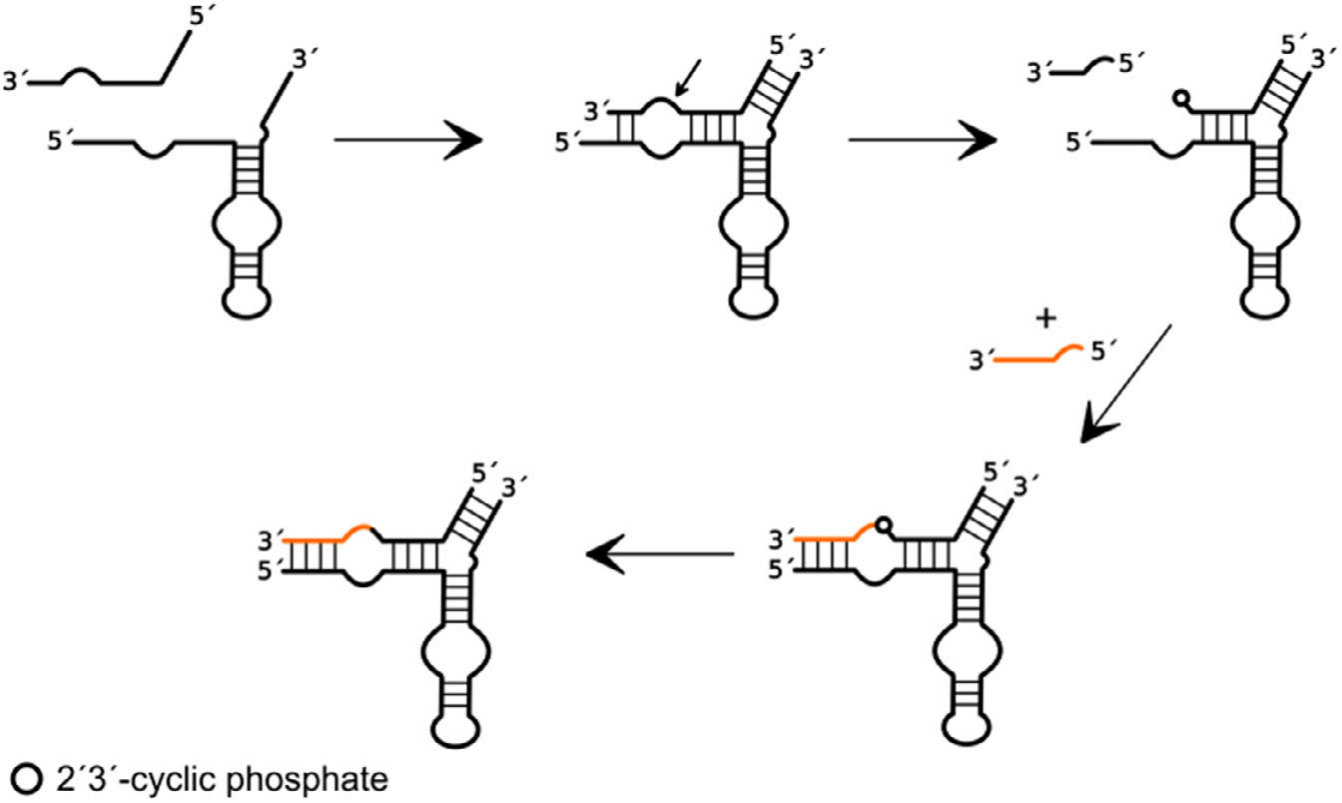
Principle of HPR‐based RNA recombination. The cleavage site is indicated by a black arrow.

The hairpin ribozyme, being well‐suited for this purpose, is a particularly advantageous starting point due to its ability to support RNA cleavage and ligation, the two essential reactions for recombination scenarios, with virtually equal efficiency. As discussed above, the relative preference for the one or the other reaction is determined by the stability of the ribozyme‐substrate complex, enabling the directed modulation of ribozyme function through structural manipulation. The protocol for HPR based recombination involves the cleavage of an RNA substrate, yielding two cleavage products: one with a 5′‐OH group and the other with a 2′,3′‐cyclic phosphate. In the specific design, the 5′‐OH‐carrying substrate is rapidly released due to its relatively loose binding to the ribozyme. Conversely, the fragment bearing the 2′,3′‐cyclic phosphate remains bound to the ribozyme, serving as the substrate for the subsequent ligation of an added ligation fragment. Due to its extended length, this fragment forms more base pairs with the ribozyme and becomes ligated, thus yielding the recombination product (**Figure** **5**). This assay has been developed in our laboratory as a tool for the preparation of long‐modified RNAs by fragment ligation (unpublished) as an alternative to classical scenarios of RNA ligation by RNA ligases.

**Figure 6:**
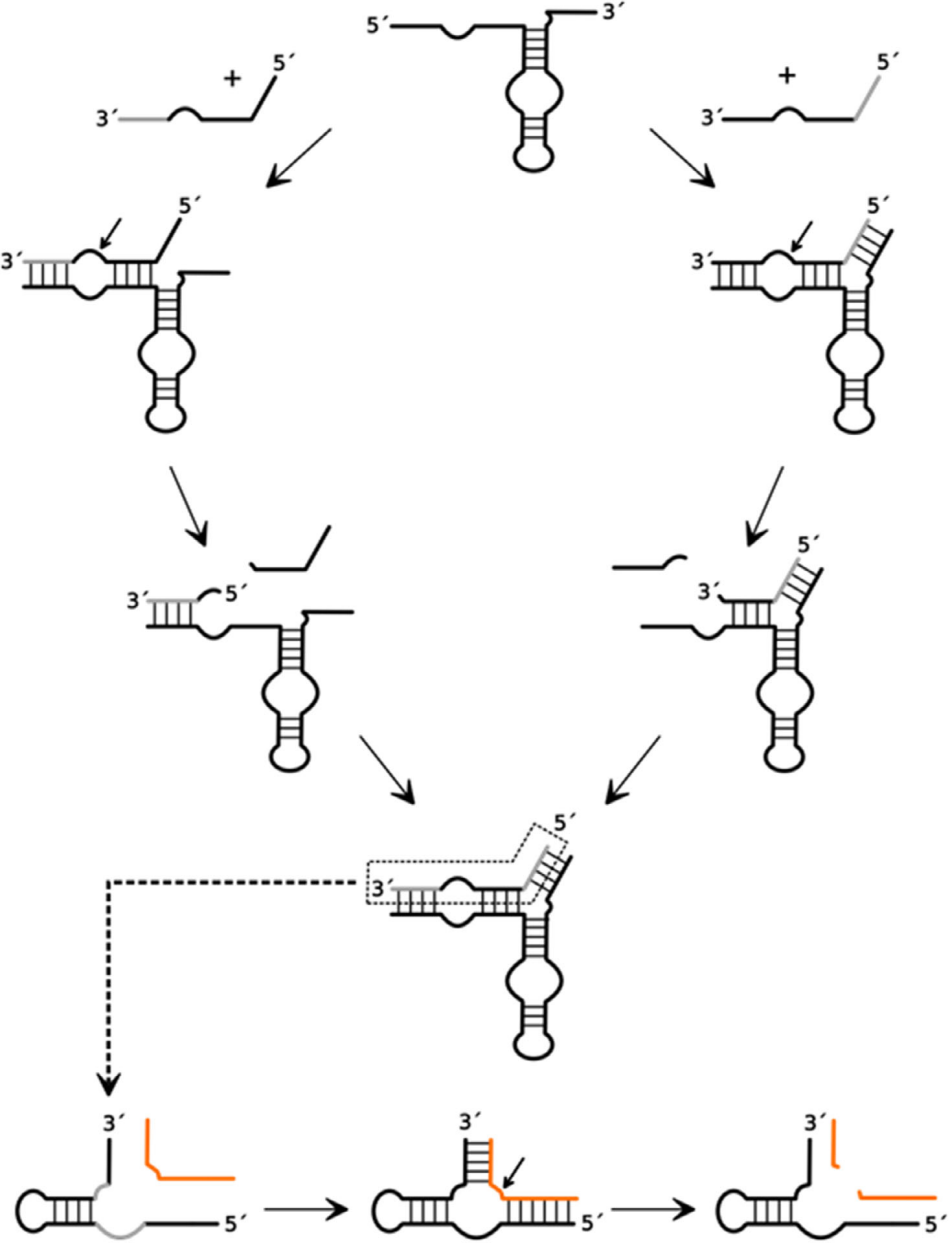
Reaction scheme of HPR based recombination to yield a functional hammerhead ribozyme. Conserved nucleotides of the HHR are marked in gray. The HHR substrate is marked in orange. Adapted with permission.^[^ ^18^ ^]^

In a different setup, we have demonstrated that HPR‐mediated recombination can be used to generate RNAs with new functionality.^[^ ^18^ ^]^ This concept involved a hairpin ribozyme variant that is capable of binding two different RNA molecules to be cleaved and ligated in recombined order, and by doing so to produce a hammerhead ribozyme as recombination product (**Figure** **6**). Consequently, in addition to the monitoring of reactions by fragment length analysis, the occurrence of successful recombination could be confirmed by employing the recombination product in a functional assay. Each of the two substrate strands provided consists of a nonfunctional and a profunctional sequence component. Subsequent to cleavage by the HPR, the nonfunctional parts are released from the HPR. The profunctional parts are designed in such a way that they are likely to rebind to the HPR to be ligated, resulting in the recombined RNA strand. The formed product itself is now an active hammerhead ribozyme able to cleave a compatible hammerhead substrate. In the initial recombination assays, the final product, that is, the hammerhead ribozyme, was obtained with only 2–9% yield. However, through extensive optimization of the experimental setup and reaction conditions (temperature, magnesium ion concentration, fragment‐ribozyme ratios), yields of up to 76% were achieved (Figure 6).^[^ ^18^ ^]^

**Figure 7:**
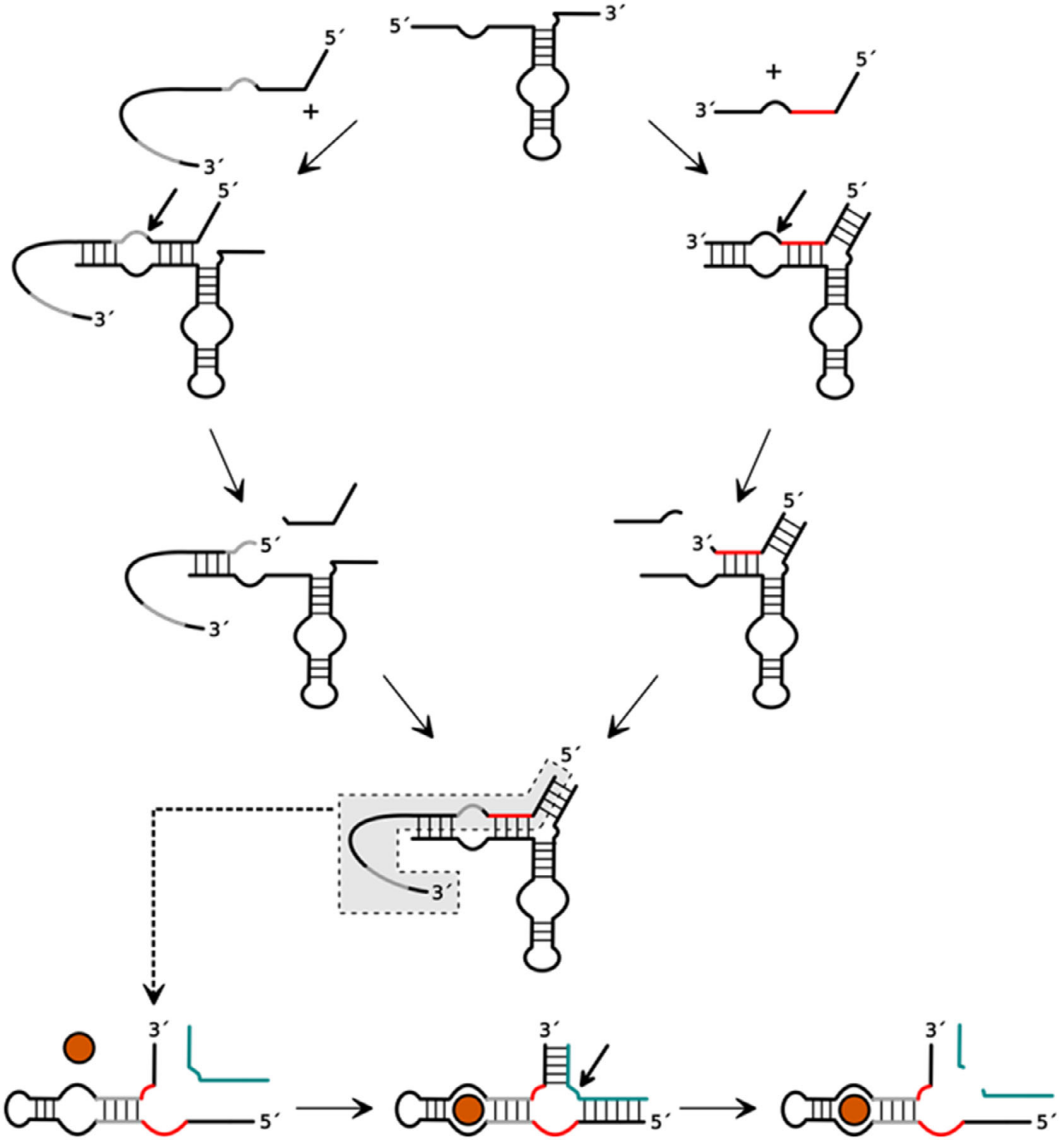
Reaction scheme of HPR‐based recombination to yield a functional aptazyme consisting of a HHR unit and a theophylline or FMN aptamer unit. HPR cleavage of the profunctional aptamer sequence is shown on the left side, and HPR cleavage of the profunctional HHR sequence is on the right. Conserved sequence parts of the HHR are marked in red. Essential sequence parts for the formation of the aptamer are marked in gray. The HHR substrate is marked in turquoise. The cognate ligand, theophylline or FMN, is represented by an orange circle. Adapted with permission.^[^ ^19^ ^]^

A similar design approach was employed for the generation of a functional aptazyme consisting of a hammerhead ribozyme unit and a theophylline or FMN aptamer unit.^[^ ^19^ ^]^ An HPR variant designed for the purpose of recombination was shown to be capable of cleaving two different RNA molecules, one containing the aptamer part and the other the hammerhead ribozyme part (**Figure** **7**). The resulting fragments were subsequently recombined and ligated to a hammerhead ribozyme that is allosterically controlled by a cognate ligand. Two such recombination processes involving aptamers for either theophylline or flavine mononucleotide (FMN) were demonstrated, yielding functional recombination products with up to 34% efficiency. Hairpin ribozyme‐mediated recombination has also been used to facilitate the assembly of RNA polymerase ribozymes^[^ ^50^ ^]^ and other long RNA molecules.^[^ ^83^ ^]^

### 4.2 Twin Ribozymes

**Figure 8:**
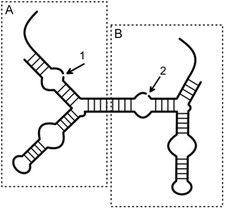
General structure of a twin ribozyme consisting of two HPR units A,B) each providing one cleavage/ligation site (1 and 2). Displayed is postcleavage ribozyme‐substrate complex before ligation.

**Figure 9:**
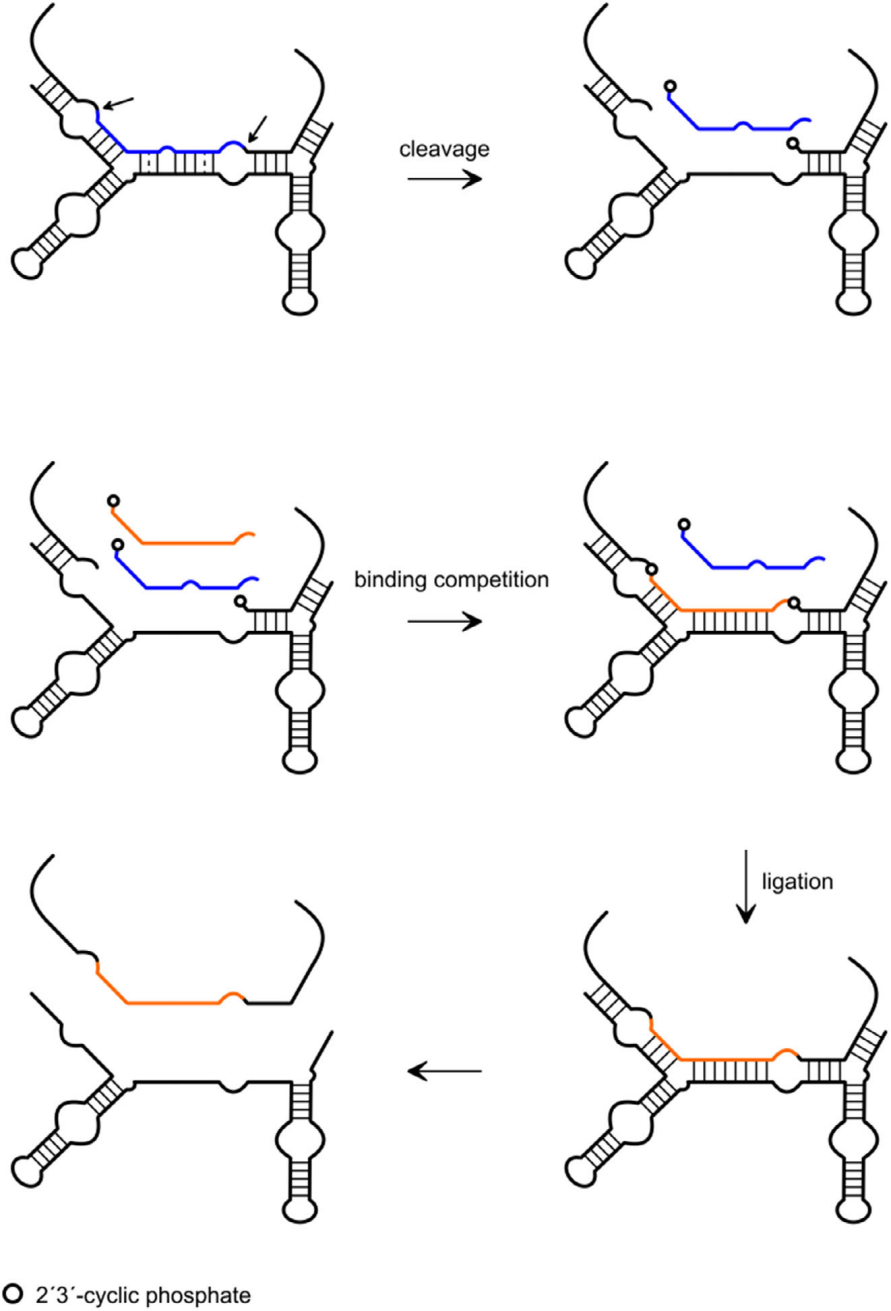
Principle of twin ribozyme mediated RNA recombination/strand exchange. Blue: substrate patch to be exchanged (with mismatches and bulge), orange: substrate patch to be inserted, white dot: 2′3′‐cyclic phosphate. Cleavage sites indicated by black arrows. Mismatches displayed as dashed lines.

One of the first projects in which we exploited the characteristic cleavage/ligation behavior of the hairpin ribozyme was the engineering of twin ribozymes to mediate an RNA fragment exchange reaction.^[^ ^17^ ^]^ In this process, a predefined section of a suitable RNA substrate is cut out and replaced by an externally added oligonucleotide. Two cleavage events and two ligations must occur in a strictly controlled manner. Twin ribozymes are derived from the hairpin ribozyme by tandem duplication (**Figure** **8**), so that two cleavage/ligation sites are generated upon binding of a suitable substrate. The basic principle of twin ribozyme action is that cleavage occurs at the two predetermined sites, producing a central fragment that can be easily dissociated from the ribozyme due to a destabilizing element such as a loop or mismatch. The two RNA fragments flanking the cleavage sites form contiguous duplexes with the ribozyme binding arms and thus remain preferentially bound to the ribozyme. The added oligonucleotide to be exchanged is designed to bind tightly to the gap left by the dissociation of the cleaved fragment, which, together with a moderate molar excess of this oligonucleotide, favors ligation and product formation (**Figure** **9**). Over the years, we have experimentally validated this in a number of scenarios where, depending on the specific design of the twin ribozyme‐substrate complex, fragments of equal length were exchanged^[^ ^73^ ^]^ or a shorter patch was replaced by a longer one^[^ ^17^, ^22^, ^73^, ^84^, ^85^ ^]^ and vice versa.^[^ ^74^ ^]^ Thus, the described scheme mimics the repair of short deletions, insertions, and base substitutions with up to 53% yield.^[^ ^84^ ^]^

Ribozyme‐mediated ligation by recombination is also a useful method for producing longer RNAs carrying site‐specific modifications. We have shown in the past that twin ribozymes also support fragment exchange with site‐specifically modified/functionalized oligonucleotides, providing an elegant approach to introduce internal modifications into large RNAs.^[^ ^73^ ^]^

### 4.3 Splicing

Another scenario where a combination of RNA cleavage and ligation is required in a scheme of subsequent reactions is RNA splicing. In biological systems today, splicing is facilitated by either group I and group II self‐splicing introns or by the spliceosome, all three of which are ribozymes that support transesterification reactions, but are quite large and complex.^[^ ^86^, ^87^, ^88^ ^]^ In the case of the spliceosome, even proteins are required for structural stabilization and regulation.

**Figure 10:**
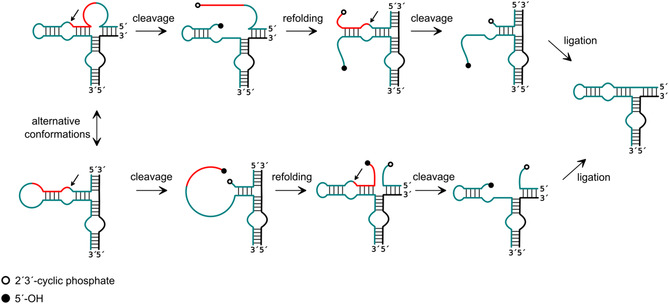
Reaction scheme for HPR‐based RNA splicing. Red: intron to be spliced out, turquoise: exon; black: supporting factor; white dot: 2′,3′‐cyclic phosphate, black dot: 5′‐OH. The cleavage sites are indicated by black arrows. Adapted with permission.^[^ ^21^ ^]^

We have recently shown that much smaller ribozymes, such as the hairpin ribozyme, are able to splice an intron between two exons, resulting in the two ligated exons and the release of the intron.^[^ ^20^, ^21^ ^]^ To mimic the exon‐intron‐exon constellation of an mRNA to be spliced, the substrate strand of the HPR was designed to contain two HPR‐specific cleavage sites. The substrate is cleaved sequentially at these two sites, releasing the sequence patch between them as an intron, after which the two exons can be ligated. To facilitate the process described with only one HPR, the overall structure of the ribozyme with its substrate must be able to adopt two alternative folds, each of which directs one of the two cleavage sites to the correct position. After the first cleavage, the ribozyme refolds into the alternative conformation so that the second cleavage can take place. After the release of the intron, the two exons carry the functionalities required for ligation at their ends and can be ligated by the ribozyme (**Figure** **10**).

It is important for the success of the splicing reaction that, firstly, the intron sequence is easily released from the HPR to prevent religation to the exons and, secondly, that the complex is able to fold into a sufficiently stable conformation after release of the intron to allow ligation. The potential folding of all reaction intermediates must therefore be carefully investigated. In one scenario, we have designed a hairpin ribozyme‐derived spliceozyme that mediates two RNA cleavages and a ligation event at specific positions, thereby excising a segment (intron) from the parent RNA and ligating the remaining fragments (exons). The excised intron then performs a downstream function, acting as a positive regulator of the activity of a bipartite DNA enzyme. To control the splicing reaction, the spliceozyme was assembled from two RNA strands: one containing the exons and the intron, and the other containing essential parts of the functional sequence necessary to reconstitute the complete ribozyme structure (supporting factor, black in Figure 10). Splicing was successful, as confirmed by fragment length analysis and in a downstream functional assay, as mentioned above, based on the presence of the excised intron as a positive regulator of the activity of a DNA enzyme.^[^ ^21^ ^]^

**Figure 11:**
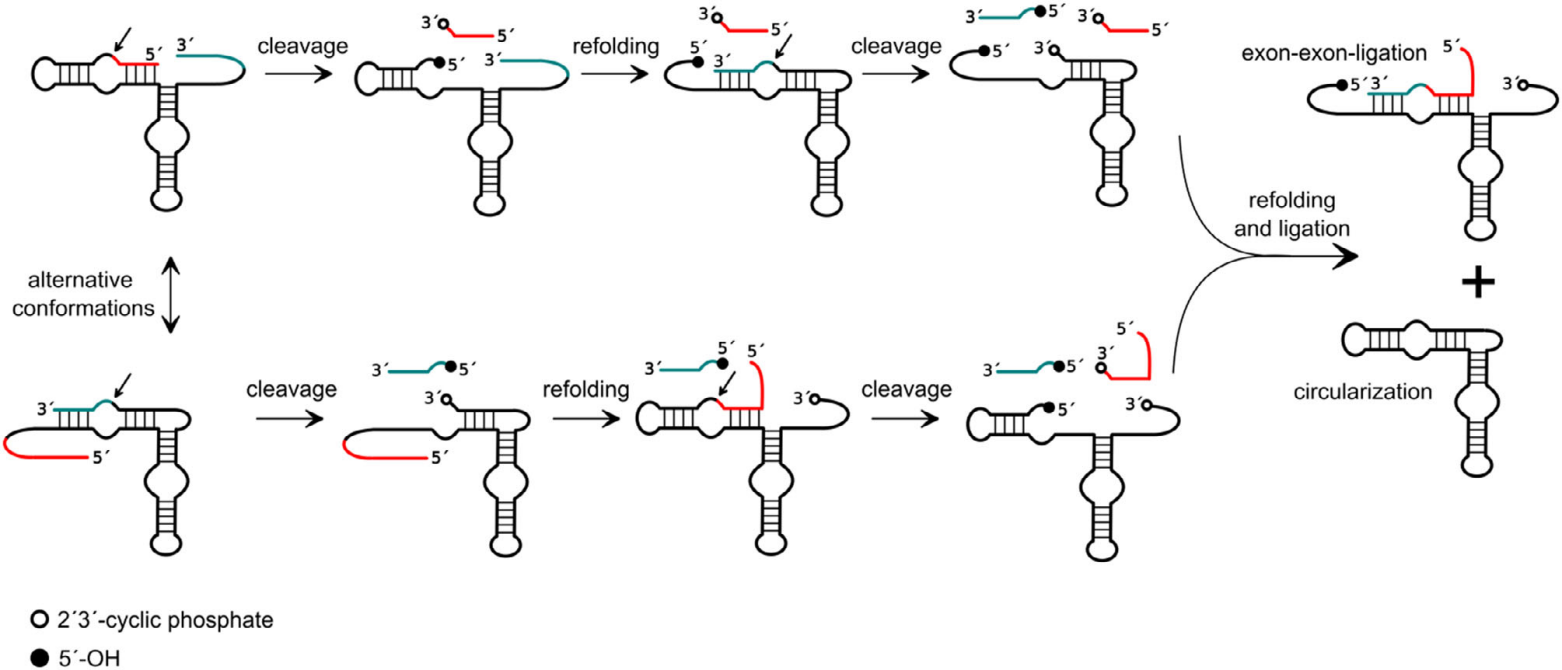
Reaction scheme for HPR‐based RNA self‐splicing. Red: 5′‐exon, turquoise: 3′‐exon; white dot: 2′,3′‐cyclophosphate, black dot: 5′‐OH, The cleavage sites are indicated by black arrows. Adapted with permission.^[^ ^20^ ^]^

In a parallel effort, we have developed another RNA splicing scenario based on the hairpin ribozyme as shown in **Figure** **11** ^[^ ^20^ ^]^ Similar to the above system, the ribozyme is able to adopt two alternative conformations, in which one of two possible cleavage sites can be cleaved. However, unlike the system described above, the activity is not located in the exonic regions, but the intron itself is the catalytic unit, analogous to the naturally self‐splicing introns. After the first cleavage in one of the two possible conformations, one of the two exons is released. The HPR can then refold into the other conformation, allowing the second cleavage to take place. After the two cleavage events and release of products, the catalytic intron is available for exon rebinding and ligation, completing the splicing process. In a competing scenario, however, the intron can refold to bring its termini, which carry the necessary functionalities (5′‐OH and 2′,3′‐cyclic phosphate), into the catalytic center and support intramolecular ligation. This competitive reaction would lead to circularization. In the original experimental setup, we had split the ribozyme strand into two parts by removing the hairpin loop at the bottom of the ribozyme, as in the example described above, in order to be able to control the reaction. Therefore, in the practical experiment, the intron ligation resulted in a linear product.

### 4.4 Circularization

The generation and function of circular RNA (circRNA) in nature have been the focus of intensive research over the past two decades. In biological systems, circRNAs are formed via an alternative process to regular splicing, called back‐splicing. Rather than joining an upstream 5′ splice site to a downstream 3′ splice site, a downstream 5′ splice site is ligated to an upstream 3′ splice site, resulting in a circular RNA.^[^ ^89^ ^]^

Building upon the finding of our splicing designs, we have developed novel RNAs that undergo a splicing reaction, but preferentially form a circular structure through intramolecular ligation of the intron rather than exon ligation.^[^ ^23^, ^24^, ^90^ ^]^ The design principle is analogous to the approach described above for the regular self‐splicing HPR. As with the previous design, two cleavage sites must be incorporated and the HPR should fold into two alternative conformations, each capable of cleaving one of the two cleavage sites. Subsequent to the first cleavage, the ribozyme undergoes a refolding process, enabling the subsequent occurrence of the second cleavage. The resultant free ends of the intron are then ligated to form a circular RNA (Figure 11). Notably, this RNA retains the active HPR sequence. Therefore, a notable disadvantage of this approach is the relative instability of the circular products attributable to their intrinsic self‐cleavage activity.

**Figure 12:**
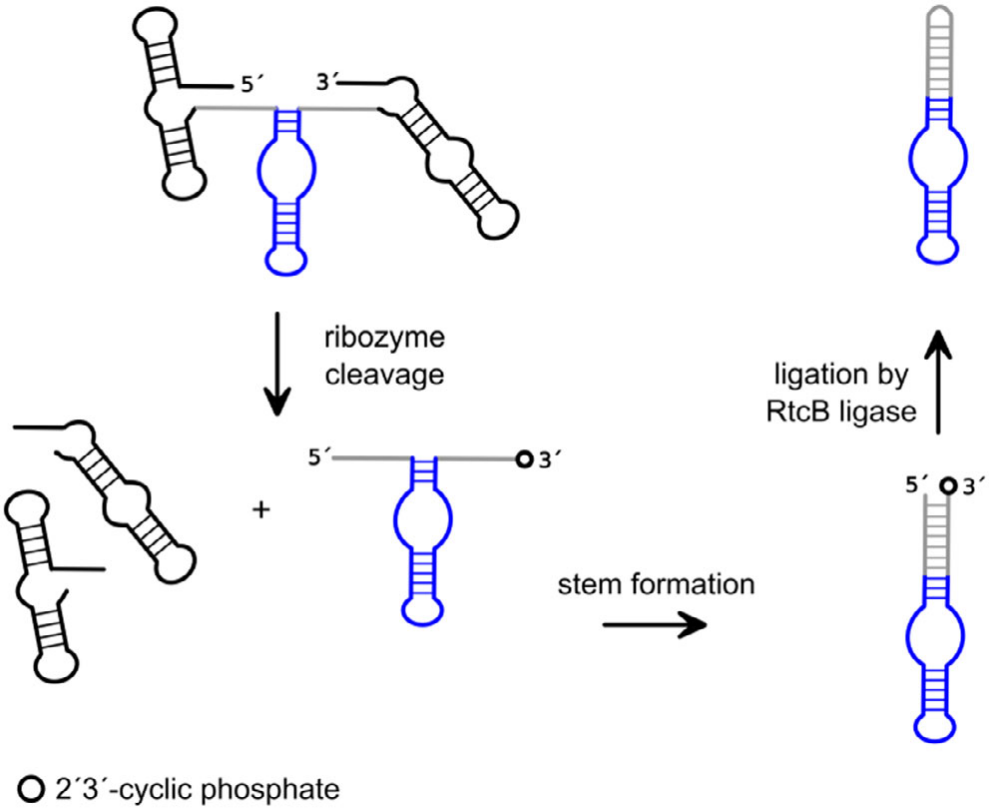
Reaction scheme for twister ribozyme‐assisted RNA circularization. Black: flanking twister ribozymes; gray: stem forming sequences; blue: RNA to be circularized; white dot: 2′,3′‐cyclic phosphate. Adapted with permission.^[^ ^91^ ^]^

An effective circularization strategy that yields more stable circular products has been developed by Jaffrey and colleagues.^[^ ^91^ ^]^ This strategy, termed the “Tornado” system (twister‐optimized RNA for durable overexpression) is also supported by ribozymes. However, the final circularization relies on the activity of a protein enzyme, the RtcB ligase. The system has been implemented for the production of circular RNA aptamers, but in principle could be used for any desired RNA sequence of interest. In the Tornado system, the RNA sequence to be circularized is flanked by engineered twister ribozymes. After transcription of the entire RNA, the incorporated ribozymes perform their specific cleavage reaction. The results of this reaction are the two twister fragments separated from the RNA of interest. Subsequent to the cleavage reaction, the RNA now possesses the end functionalities (5′‐OH and 2′,3′‐cyclic phosphate) required for the RtcB‐supported ligation reaction and can be circularized by this enzyme (**Figure** **12**). The described system not only is applicable in vitro, but has also been shown to function directly in cells, allowing the expression of circular aptamers in vivo.

In the context of the Tornado system, flanking ribozymes were selected that cleave near their 3′ or 5′ termini in order to minimize residual ribozyme sequence at the ends of the RNA of interest. Furthermore, the residual sequences between the RNA of interest and the ribozyme following cleavage were engineered to be complementary to each other, facilitating their hybridization post‐cleavage to form a stem‐like structure. This configuration facilitates the proximity of the two ends for the subsequent ligation reaction, thereby enhancing the efficiency of circularization by RtcB ligase.

## 5 Variants that Fold in Response to External Stimuli

As seen in the engineering approaches for splicing and circularization, it is possible to tune the sequence of a ribozyme so that there are different possible folding conformations for a single RNA sequence. This allows different reactions to be carried out depending on the fold adopted. The designed HPRs mentioned in Chapter 4 have adopted two alternative bi‐stable conformations due to their intrinsic folding capacity. To extend the control of ribozyme folding and thus activity, another molecule can be added as an effector to induce a conformational change. Such an engineered ribozyme is called an allosteric ribozyme. The effector molecules can be either small molecules (true allosteric control) or other RNA strands called effector oligonucleotides (allosteric‐like control).

### 5.1 Allosteric Control by Effector Oligonucleotides

Since the activity of ribozymes is highly dependent on their correct conformation, ribozyme reactions can be controlled by the addition of other RNA or DNA strands (i.e., effector oligonucleotides) that either facilitate (positive control/induction) or impede (negative control/repression) proper folding of the ribozyme by binding to a specific region of the ribozyme sequence.

Early examples of positive control of the hairpin ribozyme were developed by us^[^ ^92^ ^]^ and others,^[^ ^93^, ^94^ ^]^ in all cases using an effector oligonucleotide to reconstitute the active conformation of a formerly disabled/misfolded hairpin ribozyme. Also, the opposite scenario of negative control has been described. In this scenario, an oligonucleotide effector was employed to stabilize the ribozyme in an inactive conformation.^[^ ^94^ ^]^ The design of ribozymes capable of binding a defined oligonucleotide and being switched on or off in response can be facilitated by the use of algorithms, as demonstrated by Penchovsky and colleagues^[^ ^79^ ^]^ (see also Chapter 3.2).

**Figure 13:**
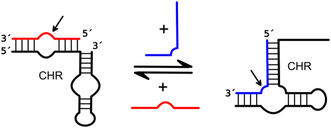
General principle of the chameleon ribozyme (CHR), which adopts either the HPR (left) or the HHR (right) fold dependent on the added substrate. Red: HPR substrate, blue: HHR substrate. The cleavage sites are indicated by black arrows. Adapted with permission.^[^ ^76^ ^]^

In a different scenario, a ribozyme can not only be modulated by the presence of an effector molecule, but also be programmed to adopt two distinct folds with different activities.^[^ ^76^, ^95^, ^96^ ^]^ Recently, we have developed an RNA capable of adopting either the HPR or HHR fold, depending on the substrate added (**Figure** **13**).

This ribozyme can adapt to its environment, which is why it's called the “chameleon ribozyme” (CHR).^[^ ^76^ ^]^ To allow this adaptability, the sequence had to be designed carefully. Unlike other engineering approaches, the structural and sequence constraints of two types of ribozymes had to be considered at the same time. Changes in the sequence to stabilize one structure must not interfere with the structure of the other, and vice versa. Thus, the design involved inspecting the minimally conserved sequences of HPR and HHR, which showed that the conserved regions of the two ribozymes were indeed compatible. Conserved positions in HPR were not conserved in HHR and vice versa. This allowed us to combine the two conserved sequences into a single RNA that could perform both functions. A similar scenario had already been suggested by Picirilli et al. who analyzed how the HHR and HPR sequences could intersect.^[^ ^96^ ^]^ For the chameleon ribozyme, we started with the HPR sequence and introduced a series of mutations to allow it to intersect with the HHR. It was also helpful to extend the minimal motifs to allow more stable folding and to include ligation of two fragments in the HPR fold in the experiments. Folding algorithms were used after each mutation or change in sequence length to determine if the folding was still correct with the two substrates. The final chameleon ribozyme was able to perform both cleavage and ligation reactions with the required substrates separately, and in a one‐pot reaction with different substrates present.

**Figure 14:**
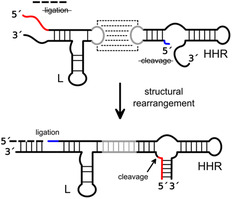
Acquisition of dual ribozyme function from two nonfunctional RNAs through kissing loop interactions. L: ligase ribozyme unit; HHR: hammerhead ribozyme unit; gray: seven‐membered loop regions; dashed line: ligase ribozyme substrate. The HHR cleavage site indicated by a black arrow. The kissing loop interaction of the two nonfunctional units causes a structural rearrangement resulting in an RNA with both ribozyme functionalities. Adapted with permission.^[^ ^97^ ^]^

Another approach to create a ribozyme with two functionalities was proposed by Tamura and colleagues.^[^ ^97^ ^]^ In contrast to the chameleon ribozyme, there was no single ribozyme strand that adopted one or the other ribozyme fold. Instead, the interaction of two separate RNA strands resulted in a catalytic RNA molecule that subsequently exhibited two ribozyme functions. For this approach, truncated nonfunctional versions of two ribozymes, the R3C ligase ribozyme and the hammerhead ribozyme, were designed, each carrying a 7‐membered single‐stranded loop region complementary to the loop of the other. As long as only one of the two strands was present, no activity could be detected. However, both strands together can interact by kissing loop interaction of the introduced 7‐membered loops. This caused a structural rearrangement resulting in a single ribozyme containing both activities, ligation by the R3C ligase and cleavage by the hammerhead ribozyme (**Figure** **14**).

### 5.2 Allosteric Control by Small Molecules

In order to exercise greater control over ribozyme reactions, it is also possible to combine two different types of functional RNAs, a ribozyme and an aptamer, in a single molecule. The resulting RNA consists of an aptamer unit (an RNA that can specifically bind a small molecule) and a ribozyme as the catalytic domain. When the small molecule binds to the aptamer, the structure of the RNA is altered, thereby enabling or disabling ribozyme activity. Depending on the design, the ribozyme unit can be allosterically controlled to be active only when a specific molecule is present or absent.^[^ ^98^ ^]^ The sequences of the two functional units can either be tandemly fused without intersecting or even overlapping to form a true aptazyme or riboswitch. After the pioneering work of Breaker and coworkers,^[^ ^98^, ^99^ ^]^ a significant number of aptazymes, predominantly those employing the hammerhead ribozyme fused to diverse aptamers, have been developed in the past.^[^ ^5^, ^15^, ^79^, ^100^ ^]^ However, alternative designs include also other ribozymes, for example an allosterically controlled Diels–Alder ribozyme that responds to theophylline.^[^ ^101^ ^]^ Often, such ribozymes are not developed through rational design but instead by in vitro selection.

**Figure 15:**
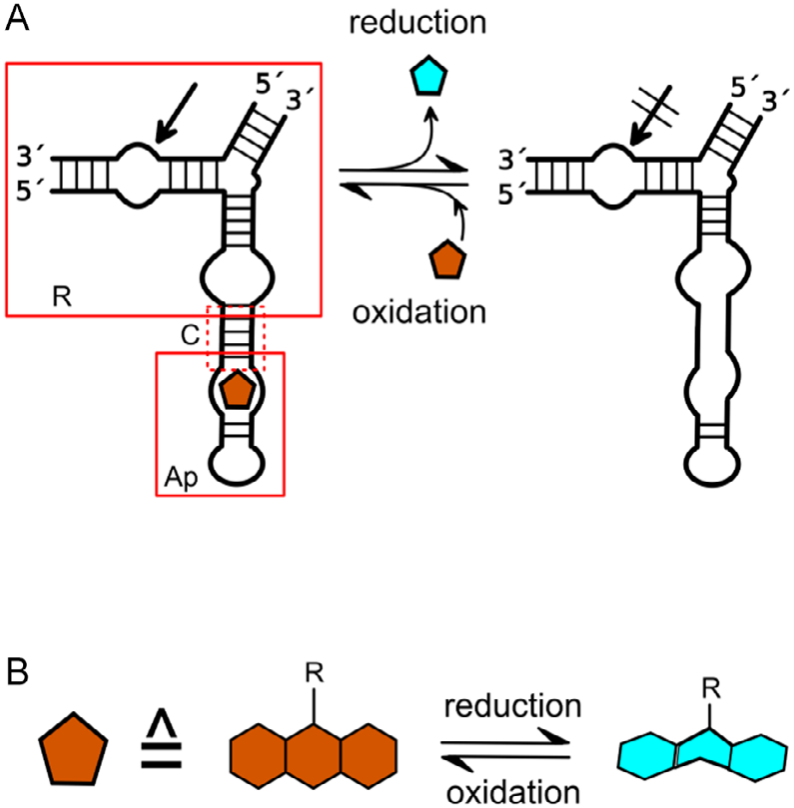
FMN‐responsive hairpin ribozyme. A) Schematic presentation of the secondary structure of an FMN‐responsive hairpin aptazyme. Binding of oxidized FMN (left) enables cleavage. After reduction of FMN, the aptamer unit cannot bind FMN and cleavage is switched off. R: ribozyme unit; Ap: FMN aptamer unit; C: communication module connecting the two units. The cleavage site indicated by a black arrow; orange pentagon: FMN. B) Conformational change of FMN upon reduction/oxidation. Adapted with permission.^[^ ^102^ ^]^

A notable example of rational design from our laboratory is the hairpin ribozyme responsive to flavine mononucleotide (FMN), which was derived by integrating the FMN aptamer into the hairpin ribozyme structure. Specifically, the HPR unit and the aptamer unit were connected by a communication module, which translates the binding event in the aptamer region to a conformational change in the ribozyme unit, thereby upregulating activity. The oxidized form of FMN is the only form that can bind to the aptamer domain, allowing the aptazyme to sense the oxidation state of its environment (**Figure** **15**).^[^ ^102^ ^]^ The reduced form of FMN exhibits much lower or no binding affinity compared to its oxidized form due to its non‐planar molecular shape. The oxidized form possesses a planar geometry due to its aromatic nature, while reduction leads to the loss of aromatic properties and results in a bent shape of the isoalloxazine ring. As a significant portion of the binding energy is derived from hydrophobic stacking of FMN with the nucleobases in the aptamer, reduction and loss of planarity cause disruption of the stacking interactions between FMN and the aptamer, leading to loss of binding. Our previous studies have demonstrated that the reduction of FMN can be achieved through either chemical or electrochemical means. More recently, we have successfully demonstrated the transport of an electron through the aptamer region, which marks the inception point for a novel design that couples FMN reduction with RNA charge transfer.^[^ ^103^ ^]^

**Figure 16:**
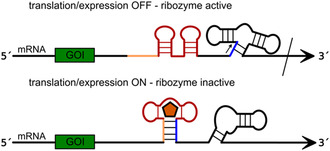
Design of an ON‐riboswitch based on the guanine aptamer and the twister ribozyme fused in tandem. In the absence of guanine (orange pentagon) the ribozyme unit is able to cleave (cleavage site indicated by black arrow) the mRNA, because the essential stem (blue) can be formed. Translation is switched OFF. If guanine binds to the aptamer unit (red), the antiribozyme sequence (orange) forms a stem with the essential strand of the ribozyme unit (blue) and the ribozyme structure is not intact anymore. Translation is switched ON. Adapted with permission.^[^ ^104^ ^]^

In another scenario, the fusion of the ribozyme with an aptamer sequence in tandem can be used for gene regulation, if the aptamer and the ribozyme sequence are included in the untranslated region (UTR) of the respective mRNA. In the case of an OFF riboswitch, the aptamer without the ligand adopts a fold that prevents the correct folding of the ribozyme moiety so that cleavage is inhibited. Conversely, in the presence of the ligand, the ribozyme undergoes a conformational change to its active form, resulting in the cleavage of the mRNA in *cis*, thereby preventing translation. In the case of an ON riboswitch, the aforementioned mechanism must function in the opposite direction, as evidenced by the study of a guanine aptamer situated in tandem with the twister ribozyme in the 5‐UTR of a mammalian mRNA.^[^ ^104^ ^]^ The absence of guanine results in the activity of the ribozyme moiety, leading to the cleavage of the mRNA. In contrast, the presence of guanine hinders the proper folding of the ribozyme, resulting in its inactivity and the subsequent translation of the mRNA (**Figure** **16**).

The engineering of this system entailed the systematic design of the aptamer‐ribozyme structure motif, aiming to adopt only two exclusive conformations. In the absence of the ligand guanine, the structure exhibited a preference for the ribozyme fold, resulting in mRNA cleavage. Conversely, in the presence of guanine, the structure predominantly adopted the aptamer fold, thereby preventing mRNA cleavage. Achieving this behavior required the prevention of proper ribozyme folding in the presence of the ligand. This was accomplished by the formation of a stem structure in the aptamer involving an essential sequence patch of the ribozyme structure (depicted in blue in Figure 16). Consequently, proper folding of the ribozyme was hindered. The design enabled the switching between the aptamer and the ribozyme structure in response to the ligand.

**Figure 17:**
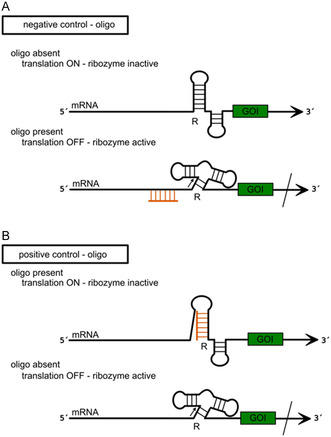
General principle of gene expression control by an effector oligonucleotide (orange) based on ribozyme (R) activity. A) Negative control: In the absence of the effector oligonucleotide the ribozyme unit folds into an inactive structure not capable of cleavage and translation is switched ON. Binding of the effector oligonucleotide to the mRNA induces a conformational change of the ribozyme unit and thereby enables mRNA cleavage (cleavage site indicated by black arrow), translation is switched OFF. B) Positive control: In presence of the effector oligonucleotide the ribozyme unit adopts an inactive fold, translation is switched ON, whereas in the absence of the effector oligonucleotide the ribozyme is active and cleaves the mRNA (cleavage site indicated by black arrow). Translation is switched OFF.

**Figure 18:**
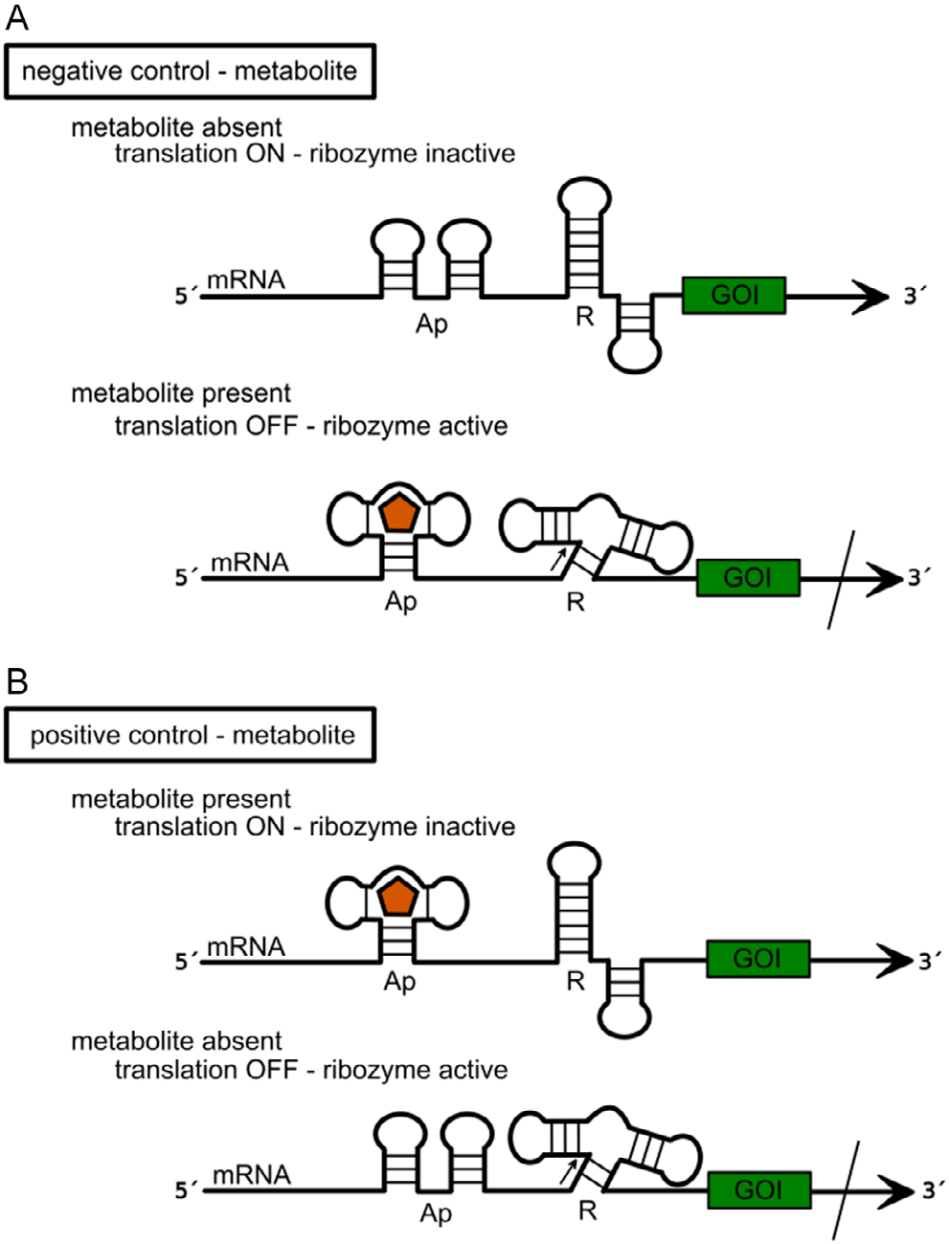
General principle of gene expression control by a small molecule (orange pentagon) based on a tandemly fused aptamer (Ap) and a ribozyme (R) unit. A) Negative control: in the absence of the small molecule, the ribozyme unit folds into an inactive structure incapable of cleavage and translation is switched ON. In the presence of the small molecule, it binds to the aptamer, which causes a conformational change of the ribozyme unit followed by mRNA cleavage (cleavage site indicated by black arrow), and translation is turned OFF. B) Positive control: Binding of the small molecule to the aptamer unit induces an inactive ribozyme fold and translation is switched ON. In the absence of the small molecule, the ribozyme adopts an active conformation and cleaves the mRNA (cleavage site indicated by black arrow). Translation is turned OFF.

Beyond the examples described above, *cis*‐acting ribozymes have been demonstrated in general to be instrumental in the regulation of gene expression across a variety of model systems.^[^ ^105^ ^]^ The underlying principle entails the integration of the ribozyme sequence into the 3′ or 5′ UTR of the mRNA that encodes the gene of interest (GOI). In the case that the ribozyme is in its active state, the mRNA is cleaved, leading to the subsequent silencing of the gene of interest. Conversely, when the ribozyme is inactivated, the mRNA remains intact and can undergo translation to produce the encoded protein (i.e., gene expression is turned on).^[^ ^106^ ^]^ The activity of the ribozyme can be modulated through the addition of a complementary oligonucleotide that binds to a specific region of the ribozyme, thereby inducing a conformational change (**Figure** **17**), or, in cases where the ribozyme is fused to an aptamer domain, by the addition of a small molecule (e.g., metabolites) (**Figure** **18**).^[^ ^106^, ^107^ ^]^ Depending on the design, it is possible to establish a negative or positive control of gene expression in response to the effector molecule. For negative control (knock‐out in the presence of the effector molecule), the ribozyme must fold into an inactive conformation after transcription of the mRNA. Only with the addition of the effector is it able to fold correctly and cleave the mRNA.^[^ ^108^, ^109^, ^110^, ^111^, ^112^ ^]^ Conversely, in a positive control scenario, where gene expression is induced by the presence of the effector molecule, the ribozyme is engineered to prevent the formation of the active conformation upon binding of the effector (Figure 17 and 18).^[^ ^104^, ^113^, ^114^ ^]^

A more recent example of positive control of gene expression involves the insertion of a variant of the hammerhead ribozyme into the 3′‐UTR of the mRNA to be controlled. Following transcription, the ribozyme cleaves the mRNA, thereby inhibiting translation. In the presence of an antisense oligonucleotide, which binds to the 3′‐UTR proximal to the ribozyme, a conformational change is induced rendering the ribozyme inactive. Consequently, translation was initiated. As this system could be applied in living organisms such as the nematode *C. elegans*, it shows that *cis*‐acting ribozymes can be a useful tool for regulating gene expression in molecular biology, but eventually also for diagnostic and therapeutic purposes.^[^ ^115^ ^]^

## 6 Ribozyme Design for Diagnostic/Therapeutic Applications

In comparison to proteins, small self‐cleaving ribozymes are more compact, and the synthesis and purification of the RNA strands are more cost‐effective and less labor‐intensive. Moreover, proteins must be expressed in cell culture, which carries the risk of contamination from the organism used for production. As proteins themselves have the potential to be immunogenic, they are not suitable for all applications. Conversely, in vitro transcribed RNA does not carry the risk of contamination from cell culture. While transcribed RNA itself can act as an immunogen, it tends to be less immunogenic than proteins and can be modified to reduce immunogenicity.^[^ ^116^, ^117^, ^118^, ^119^, ^120^, ^121^ ^]^ Due to these advantages and the relatively simple mechanism, ribozymes are suitable for diagnostic or therapeutic applications. One of the major applications is probably ribozyme‐supported mRNA cleavage to control gene expression, as discussed above. From the therapeutic perspective, this strategy is useful for the purpose of regulating the expression of mRNAs that are administered exogenously into living cells, for example in the context of anticancer treatment. As a helpful design tool, ribozymes as potential drug candidates could also be designed with the help of computational approaches.^[^ ^79^ ^]^

**Figure 19:**
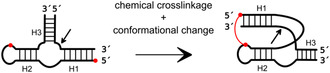
Design of hammerhead ribozyme that can be chemically crosslinked to stabilize the ribozyme in its active bent conformation. The cleavage site is indicated by a black arrow. Red dots: functional groups (i.e., azide and alkyne) for chemical crosslinking. Adapted with permission.^[^ ^124^ ^]^

Due to its relatively short sequence and compact structure combined with a high variability of possible substrate sequences, the hammerhead ribozyme is a promising candidate for diagnostic or therapeutic applications, in vitro and in cells, although there are a number of hurdles to overcome including delivery, endosomal escape, and intracellular stability.^[^ ^122^, ^123^ ^]^ In addition to these challenges common to all oligonucleotide drugs, ribozymes must be designed to be sufficiently active under cellular conditions. For example, the minimal HHR demonstrates suboptimal cleavage efficiency and requires relatively high magnesium ion concentrations, because it lacks the loop–loop interaction, present in the full‐length wild‐type HHR. A recent study of ribozyme engineering reported a clever approach to overcome this challenge and combine the advantages of the wild‐type (lower magnesium dependence, higher activity) and the minimal motif (wider range of possible substrate sequences) in one ribozyme.^[^ ^124^ ^]^ The design of a minimal HHR devoid of loop–loop interaction was accomplished, and upon folding, the ribozyme was artificially locked in the adopted conformation by chemical crosslinking (**Figure** **19**). This was achieved by cycloaddition between azide and alkyne functionalities positioned at specific sites of the ribozyme structure, thereby providing greater folding stability and thus higher activity even under physiological conditions and inside cells. As the ribozyme still corresponded to the minimal motif and did not require the additional loop for tertiary stabilization, the broad substrate range was retained. This method was further refined to develop hammerhead ribozymes, which have found application as RNA interference tools. They have been shown to cleave a specific RNA substrate, thereby inducing efficient gene silencing.^[^ ^124^ ^]^

## 7 Conclusion

The development of nucleic acid catalysts by rational design is a powerful tool with potential applications. However, it can be a very challenging task, requiring careful consideration of a range of factors related to sequence fitting, folding, and reaction equilibria. Typically, the design process begins with a precursor ribozyme that has known structural and catalytic properties. As mentioned earlier, one of the most important steps is to adapt the ribozyme sequence to recognize a defined target and to maintain activity. The substrate association and dissociation can be influenced by altering the length of the substrate‐binding domains. However, the ribozyme must still be able to fold into the required active conformation. Furthermore, structural modulation of the chosen precursor ribozyme can be used to bias the reaction equilibrium (e.g., in the hairpin ribozyme, structural stabilization favors ligation, while destabilization favors cleavage).

Numerous tools are available to facilitate rational design, including software and platforms for the theoretical prediction of secondary structures of nucleic acids, which can guide sequence design. A prerequisite for successful engineering is the understanding of the structure and mechanistic properties of the starting nucleic acid. The more data available, the higher the probability of a successful design. Currently, several nucleic acids meet this requirement. The numerous examples of ribozyme engineering demonstrate that we now possess a sufficient understanding of these systems to design them for specific applications. The hairpin ribozyme variants described here illustrate the strong relationship between the structure and function of this catalytic RNA. Through structural manipulation, variants with new properties have been created while retaining the basic functionalities, RNA cleavage and ligation. These however, were tuned to support diverse RNA processing pathways. It is noteworthy that the rational design also of other ribozymes has led to the development of novel nucleic acid catalysts with unique properties. As tools for predicting and modeling RNA structures continue to improve, RNA engineering, and in particular, the rational design of functional nucleic acids, will become less challenging. Nevertheless, these endeavors will remain a rewarding task for the development of novel RNAs for demanding applications.

## Conflict of Interest

The authors declare no conflict of interest.

## References

[1] J. Liu , Z. Cao , Y. Lu , Chem. Rev. 2009, 109, 1948. 19301873 10.1021/cr030183i PMC2681788

[2] A. Sett , S. Das , U. Bora , Appl. Biochem. Biotechnol. 2014, 174, 1073. 24903959 10.1007/s12010-014-0990-3

[3] R. M. Jimenez , J. A. Polanco , A. Luptak , Trends Biochem. Sci. 2015, 40, 648. 26481500 10.1016/j.tibs.2015.09.001 PMC4630146

[4] H. Peng , B. Latifi , S. Muller , A. Luptak , I. A. Chen , RSC Chem. Biol. 2021, 2, 1370. 34704043 10.1039/d0cb00207k PMC8495972

[5] J. Frommer , B. Appel , S. Müller , Curr. Opin. Biotechnol. 2015, 31, 35. 25146171 10.1016/j.copbio.2014.07.009

[6] R. Hieronymus , S. Müller , Ann. N. Y. Acad. Sci. 2019, 1447, 135. 30941784 10.1111/nyas.14052

[7] A. Jaschke , Curr. Opin. Struct. Biol. 2001, 11, 321. 11406381 10.1016/s0959-440x(00)00208-6

[8] M. Hollenstein , Molecules 2015, 20, 20777. 26610449 10.3390/molecules201119730 PMC6332124

[9] F. Calvanese , M. Weigt , P. Nghe , Methods Mol. Biol. 2025, 2847, 217. 39312147 10.1007/978-1-0716-4079-1_15

[10] A. Ren , R. Micura , D. J. Patel , Curr. Opin. Chem. Biol. 2017, 41, 71. 29107885 10.1016/j.cbpa.2017.09.017 PMC7955703

[11] M. Egger , R. Bereiter , S. Mair , R. Micura , Angew. Chem., Int. Ed. 2022, 61, e202207590. 10.1002/anie.202207590 PMC9826390 35982640

[12] S. Muller , B. Appel , D. Balke , R. Hieronymus , C. Nubel , F1000 Res. 2016, 5, 1511. 10.12688/f1000research.8601.1 PMC4926735 27408700

[13] R. Chen , S. K. Wang , J. A. Belk , L. Amaya , Z. Li , A. Cardenas , B. T. Abe , C. K. Chen , P. A. Wender , H. Y. Chang , Nat. Biotechnol. 2023, 41, 262. 35851375 10.1038/s41587-022-01393-0 PMC9931579

[14] S. Umekage , Y. Kikuchi , J. Biotechnol. 2009, 139, 265. 19138712 10.1016/j.jbiotec.2008.12.012

[15] M. Felletti , J. S. Hartig , Wiley Interdiscip. Rev.: RNA 2017, 8, e1395. 10.1002/wrna.1395 27687155

[16] S. V. Park , J. S. Yang , H. Jo , B. Kang , S. S. Oh , G. Y. Jung , R. N. A. Catalytic , Biotechnol. Adv. 2019, 37, 107452. 31669138 10.1016/j.biotechadv.2019.107452

[17] R. Welz , K. Bossmann , C. Klug , C. Schmidt , H. J. Fritz , S. Muller , Angew. Chem., Int. Ed. 2003, 42, 2424. 10.1002/anie.200250611 12783515

[18] R. Hieronymus , S. P. Godehard , D. Balke , S. Müller , Chem. Commun. 2016, 52, 4365. 10.1039/c6cc00383d 26923676

[19] R. Hieronymus , S. Müller , ChemSysChem 2021, 3, 2100003.

[20] R. Hieronymus , J. Zhu , S. Müller , Nucleic Acids Res. 2022, 50, 368. 34928378 10.1093/nar/gkab1239 PMC8754997

[21] J. Zhu , R. Hieronymus , S. Müller , ChemBioChem 2023, 24, 475. 10.1002/cbic.202300204 37184100

[22] D. Balke , I. Zieten , A. Strahl , O. Müller , S. Müller , ChemMedChem 2014, 9, 2128. 25112518 10.1002/cmdc.201402166

[23] S. Petkovic , S. Müller , FEBS Lett. 2013, 587, 2435. 23796421 10.1016/j.febslet.2013.06.013

[24] S. Petkovic , S. Badelt , S. Block , C. Flamm , M. Delcea , I. Hofacker , S. Müller , RNA 2015, 21, 1249. 25999318 10.1261/rna.047670.114 PMC4478344

[25] A. C. Forster , R. H. Symons , Cell 1987, 50, 9. 3594567 10.1016/0092-8674(87)90657-x

[26] J. Deng , Y. Shi , X. Peng , Y. He , X. Chen , M. Li , X. Lin , W. Liao , Y. Huang , T. Jiang , D. M. J. Lilley , Z. Miao , L. Huang , Nucleic Acids Res. 2023, 51, D262. 36177882 10.1093/nar/gkac840 PMC9825448

[27] D. M. J. Lilley , Philos. Trans. R. Soc. London, Ser. B 2011, 366, 2910. 21930582 10.1098/rstb.2011.0132 PMC3158914

[28] R. Hanna , J. A. Doudna , Curr. Opin. Chem. Biol. 2000, 4, 166. 10742186 10.1016/s1367-5931(99)00071-x

[29] V. K. Misra , D. E. Draper , Biopolymers 1998, 48, 113. 10333741 10.1002/(SICI)1097-0282(1998)48:2<113::AID-BIP3>3.0.CO;2-Y

[30] R. Yamagami , J. P. Sieg , P. C. Bevilacqua , Biochemistry 2021, 60, 2374. 34319696 10.1021/acs.biochem.1c00012 PMC8747768

[31] J. M. Buzayan , W. L. Gerlach , G. Bruening , Nature 1986, 323, 349.

[32] S. Alam , V. Grum‐Tokars , J. Krucinska , M. L. Kundracik , J. E. Wedekind , Biochemistry 2005, 44, 14396. 16262240 10.1021/bi051550i

[33] P. B. Rupert , A. R. Ferré‐D’Amaré , Nature 2001, 410, 780. 11298439 10.1038/35071009

[34] S. Müller , B. Appel , T. Krellenberg , S. Petkovic , IUBMB Life 2012, 64, 36. 22131309 10.1002/iub.575

[35] S. E. Butcher , J. M. Burke , J. Mol. Biol. 1994, 244, 52. 7966321 10.1006/jmbi.1994.1703

[36] S. Joseph , A. Berzal‐Herranz , B. M. Chowrira , S. E. Butcher , J. M. Burke , Genes Dev. 1993, 7, 130. 7678568 10.1101/gad.7.1.130

[37] A. Berzal‐Herranz , S. Joseph , B. M. Chowrira , S. E. Butcher , J. M. Burke , EMBO J. 1993, 12, 2567. 8508779 10.1002/j.1460-2075.1993.tb05912.x PMC413496

[38] R. Pinard , D. Lambert , N. G. Walter , J. E. Heckman , F. Major , J. M. Burke , Biochemistry 1999, 38, 16035. 10587425 10.1021/bi992024s

[39] M. J. Fedor , J. Mol. Biol. 2000, 297, 269. 10715200 10.1006/jmbi.2000.3560

[40] D. J. Earnshaw , M. L. Hamm , J. A. Piccirilli , A. Karpeisky , L. Beigelman , B. S. Ross , M. Manoharan , M. J. Gait , Biochemistry 2000, 39, 6410. 10828955 10.1021/bi992974d

[41] M. J. Fedor , Biochemistry 1999, 38, 11040. 10460159 10.1021/bi991069q

[42] X. Zhuang , H. Kim , M. J. Pereira , H. P. Babcock , N. G. Walter , S. Chu , Science 2002, 296, 1473. 12029135 10.1126/science.1069013

[43] B. P. Paudel , D. Rueda , J. Am. Chem. Soc. 2014, 136, 16700. 25399908 10.1021/ja5073146 PMC4277754

[44] R. Pinard , K. J. Hampel , J. E. Heckman , D. Lambert , P. A. Chan , F. Major , J. M. Burke , EMBO J. 2001, 20, 6434. 11707414 10.1093/emboj/20.22.6434 PMC125305

[45] M. K. Nahas , T. J. Wilson , S. C. Hohng , K. Jarvie , D. M. J. Lilley , T. Ha , Nat. Struct. Mol. Biol. 2004, 11, 1107. 15475966 10.1038/nsmb842

[46] R. Welz , C. Schmidt , S. Muller , Biochem. Biophys. Res. Commun. 2001, 283, 648. 11341774 10.1006/bbrc.2001.4829

[47] N. G. Walter , J. M. Burke , D. P. Millar , Nat. Struct. Biol. 1999, 6, 544. 10360357 10.1038/9316

[48] J. B. Murray , A. A. Seyhan , N. G. Walter , J. M. Burke , W. G. Scott , Chem. Biol. 1998, 5, 587. 9818150 10.1016/s1074-5521(98)90116-8

[49] A. V. Vlassov , B. H. Johnston , S. A. Kazakov , Oligonucleotides 2005, 15, 303. 16396624 10.1089/oli.2005.15.303

[50] H. Mutschler , A. Wochner , P. Holliger , Nat. Chem. 2015, 7, 502. 25991529 10.1038/nchem.2251 PMC4495579

[51] T. J. Wilson , M. Nahas , L. Araki , S. Harusawa , T. Ha , D. M. J. Lilley , Blood Cells, Mol., Dis. 2007, 38, 8. 17150385 10.1016/j.bcmd.2006.10.004

[52] N. Usman , L. Beigelman , J. A. McSwiggen , Curr. Opin. Struct. Biol. 1996, 6, 527. 8794164 10.1016/s0959-440x(96)80119-9

[53] G. A. Prody , J. T. Bakos , J. M. Buzayan , I. R. Schneider , G. Bruening , Science 1986, 231, 1577. 17833317 10.1126/science.231.4745.1577

[54] H. W. Pley , K. M. Flaherty , D. B. McKay , Nature 1994, 372, 68. 7969422 10.1038/372068a0

[55] K. R. Birikh , P. A. Heaton , F. Eckstein , Eur. J. Biochem. 1997, 245, 1. 9128718 10.1111/j.1432-1033.1997.t01-3-00001.x

[56] M. Martick , W. G. Scott , Cell 2006, 126, 309. 16859740 10.1016/j.cell.2006.06.036 PMC4447102

[57] M. D. La Peña , S. Gago , R. Flores , EMBO J. 2003, 22, 5561. 14532128 10.1093/emboj/cdg530 PMC213784

[58] W. G. Scott , L. H. Horan , M. Martick , Prog. Mol. Biol. Transl. Sci. 2013, 120, 1. 24156940 10.1016/B978-0-12-381286-5.00001-9 PMC4008931

[59] C. Hammann , A. Luptak , J. Perreault , M. D. La Peña , RNA 2012, 18, 871. 22454536 10.1261/rna.031401.111 PMC3334697

[60] K. J. Hertel , O. C. Uhlenbeck , Biochemistry 1995, 34, 1744. 7849034 10.1021/bi00005a031

[61] K. J. Hertel , D. Herschlag , O. C. Uhlenbeck , Biochemistry 1994, 33, 3374. 8136375 10.1021/bi00177a031

[62] T. K. Stage‐Zimmermann , O. C. Uhlenbeck , RNA 1998, 4, 875. 9701280 10.1017/s1355838298980876 PMC1369666

[63] T. K. Stage‐Zimmermann , O. C. Uhlenbeck , Nat. Struct. Biol. 2001, 8, 863. 11573091 10.1038/nsb1001-863

[64] K. F. Blount , O. C. Uhlenbeck , Biochemistry 2002, 41, 6834. 12022888 10.1021/bi025596c

[65] J. A. Nelson , I. Shepotinovskaya , O. C. Uhlenbeck , Biochemistry 2005, 44, 14577. 16262257 10.1021/bi051130t

[66] J. Brill , C. Nurmi , Y. F. Li , ChemBioChem 2024, 25, e202400432. 39116094 10.1002/cbic.202400432

[67] A. V. Vlassov , B. H. Johnston , L. F. Landweber , S. A. Kazakov , Nucleic Acids Res. 2004, 32, 2966. 15161960 10.1093/nar/gkh601 PMC419604

[68] S. A. Kazakov , S. V. Balatskaya , B. H. Johnston , RNA 2006, 12, 446. 16495237 10.1261/rna.2123506 PMC1383583

[69] J. Schnabl , R. K. O. Sigel , Curr. Opin. Chem. Biol. 2010, 14, 269. 20047851 10.1016/j.cbpa.2009.11.024

[70] S. DasGupta , Org. Biomol. Chem. 2020, 18, 7724. 32914154 10.1039/d0ob01695k

[71] S. DasGupta , S. Zhang , J. W. Szostak , ACS Cent. Sci. 2023, 9, 1670. 37637737 10.1021/acscentsci.3c00547 PMC10451029

[72] I. Drude , A. Strahl , D. Galla , O. Müller , S. Müller , FEBS J. 2011, 278, 622. 21199369 10.1111/j.1742-4658.2010.07983.x

[73] S. Vauleon , S. A. Ivanov , S. Gwiazda , S. Muller , ChemBioChem 2005, 6, 2158. 16276501 10.1002/cbic.200500215

[74] I. Drude , S. Vauleon , S. Muller , Biochem. Biophys. Res. Commun. 2007, 363, 24. 17825791 10.1016/j.bbrc.2007.08.135

[75] Y. Komatsu , M. Shirai , S. Yamashita , E. Ohtsuka , Bioorg. Med. Chem. 1997, 5, 1063. 9222499 10.1016/s0968-0896(97)00042-4

[76] J. Zhu , D. Dierks , C. Möller , D. Balke , S. Müller , Angew. Chem., Int. Ed. 2024, 63, e202409047. 10.1002/anie.202409047 38940693

[77] M. Daher , A. M. Mustoe , A. Morriss‐Andrews , C. L. Brooks , N. G. Walter , Nucleic Acids Res. 2017, 45, 9706. 28934478 10.1093/nar/gkx614 PMC5766210

[78] S. Najeh , K. Zandi , N. Kharma , J. Perreault , RNA 2023, 29, 764. 36868786 10.1261/rna.079148.122 PMC10187678

[79] D. Kaloudas , N. Pavlova , R. Penchovsky , J. Biotechnol. 2023, 373, 82. 37499876 10.1016/j.jbiotec.2023.07.005

[80] J. Sarzynska , M. Popenda , M. Antczak , M. Szachniuk , Proteins: Struct., Funct., Bioinf. 2023, 91, 1790. 10.1002/prot.26578 37615316

[81] M. Chaturvedi , M. A. Rashid , K. K. Paliwal , Comput. Biol. Med. 2025, 188, 109845. 39983363 10.1016/j.compbiomed.2025.109845

[82] J. Wang , Y. Fan , L. Hong , Z. Hu , Y. Li , Curr. Opin. Struct. Biol. 2025, 91, 102991. 39933218 10.1016/j.sbi.2025.102991

[83] K. Le Vay , E. Y. Song , B. Ghosh , T. D. Tang , H. Mutschler , Angew. Chem., Int. Ed. 2021, 60, 26096. 10.1002/anie.202109267 PMC9299051 34569680

[84] D. Balke , A. Becker , S. Müller , Org. Biomol. Chem. 2016, 14, 6729. 27314882 10.1039/c6ob01043a

[85] S. A. Ivanov , S. Vauleon , S. Muller , FEBS J. 2005, 272, 4464. 16128815 10.1111/j.1742-4658.2005.04865.x

[86] T. R. Cech , Annu. Rev. Biochem. 1990, 59, 543. 2197983 10.1146/annurev.bi.59.070190.002551

[87] K. Lehmann , U. Schmidt , Crit. Rev. Biochem. Mol. Biol. 2003, 38, 249. 12870716 10.1080/713609236

[88] C. L. Will , R. Lührmann , Cold Spring Harbor Perspect. Biol. 2011, 3, a003707. 10.1101/cshperspect.a003707 PMC3119917 21441581

[89] J. Salzman , C. Gawad , P. L. Wang , N. Lacayo , P. O. Brown , PLoS One 2012, 7, e30733. 22319583 10.1371/journal.pone.0030733 PMC3270023

[90] S. Pieper , S. Vauleon , S. Muller , Biol. Chem. 2007, 388, 743. 17570827 10.1515/BC.2007.067

[91] J. L. Litke , S. R. Jaffrey , Nat. Biotechnol. 2019, 37, 667. 30962542 10.1038/s41587-019-0090-6 PMC6554452

[92] S. Vauléon , S. Müller , ChemBioChem 2003, 4, 220. 12616637 10.1002/cbic.200390035

[93] Y. Komatsu , K. Nobuoka , N. Karino‐Abe , A. Matsuda , E. Ohtsuka , Biochemistry 2002, 41, 9090. 12119023 10.1021/bi020012s

[94] S. H. Najafi‐Shoushtari , Nucleic Acids Res. 2004, 32, 3212. 15199169 10.1093/nar/gkh643 PMC434448

[95] E. A. Schultes , D. P. Bartel , Science 2000, 289, 448. 10903205 10.1126/science.289.5478.448

[96] S. DasGupta , K. Nykiel , J. A. Piccirilli , RNA 2021, 27, 1017. 34131025 10.1261/rna.078813.121 PMC8370743

[97] H. Mutsuro‐Aoki , K. Tamura , Life 2022, 12, 1561. 36294996 10.3390/life12101561 PMC9604999

[98] J. Tang , R. R. Breaker , Chem. Biol. 1997, 4, 453. 9224568 10.1016/s1074-5521(97)90197-6

[99] G. A. Soukup , R. R. Breaker , Proc. Natl. Acad. Sci. U. S. A. 1999, 96, 3584. 10097080 10.1073/pnas.96.7.3584 PMC22337

[100] M. Araki , Y. Okuno , Y. Hara , Y. Sugiura , Nucleic Acids Res. 1998, 26, 3379. 9649622 10.1093/nar/26.14.3379 PMC147720

[101] M. Helm , M. Petermeier , B. Ge , R. Fiammengo , A. Jaschke , J. Am. Chem. Soc. 2005, 127, 10492. 16045328 10.1021/ja052886i

[102] D. Strohbach , N. Novak , S. Müller , Angew. Chem., Int. Ed. 2006, 45, 2127. 10.1002/anie.200503820 16502442

[103] J. Frommer , S. Muller , Angew. Chem., Int. Ed. 2020, 59, 22999. 10.1002/anie.202009430 PMC7756803 32852119

[104] K. Mustafina , K. Fukunaga , Y. Yokobayashi , ACS Synth. Biol. 2020, 9, 19. 31820936 10.1021/acssynbio.9b00371

[105] D. Kläge , E. Müller , J. S. Hartig , RNA Biol. 2024, 21, 210. 10.1080/15476286.2023.2296203 PMC10761166 38146121

[106] Y. Yokobayashi , Curr. Opin. Chem. Biol. 2019, 52, 72. 31238268 10.1016/j.cbpa.2019.05.018 PMC7108311

[107] M. Spöring , M. Finke , J. S. Hartig , Curr. Opin. Biotechnol. 2020, 63, 34. 31811992 10.1016/j.copbio.2019.11.008

[108] G. Zhong , H. Wang , C. C. Bailey , G. Gao , M. Farzan , eLife 2016, 5, e18858. 27805569 10.7554/eLife.18858 PMC5130294

[109] Y. Nomura , L. Zhou , A. Miu , Y. Yokobayashi , ACS Synth. Biol. 2013, 2, 684. 23697539 10.1021/sb400037a PMC3874218

[110] N. Shanidze , F. Lenkeit , J. S. Hartig , D. Funck , Plant Physiol. 2020, 182, 123. 31704721 10.1104/pp.19.00625 PMC6945857

[111] K. Takahashi , Y. Yokobayashi , ACS Synth. Biol. 2019, 8, 1976. 31415142 10.1021/acssynbio.9b00177

[112] B. Strobel , B. Klauser , J. S. Hartig , T. Lamla , F. Gantner , S. Kreuz , Mol. Ther. 2015, 23, 1582. 26137851 10.1038/mt.2015.123 PMC4817922

[113] A. Ogawa , M. Maeda , ChemBioChem 2008, 9, 206. 18098257 10.1002/cbic.200700478

[114] K. Mustafina , Y. Nomura , R. Rotrattanadumrong , Y. Yokobayashi , ACS Synth. Biol. 2021, 10, 2040. 34374523 10.1021/acssynbio.1c00213

[115] J. Fang , J. Wang , Y. Wang , X. Liu , B. Chen , W. Zou , Commun. Biol. 2023, 6, e00471. 10.1038/s42003-023-05184-4 PMC10403566 37542105

[116] M. Wolczyk , J. Szymanski , I. Trus , Z. Naz , T. Tame , A. Bolembach , N. R. Choudhury , K. Kasztelan , J. Rappsilber , A. Dziembowski , G. Michlewski , Nucleic Acids Res. 2024, 53, gkae1252. 10.1093/nar/gkae1252 PMC11797061 39704128

[117] X. Mu , S. Hur , Acc. Chem. Res. 2021, 54, 4012. 34677064 10.1021/acs.accounts.1c00521 PMC9127547

[118] X. Mu , E. Greenwald , S. Ahmad , S. Hur , Nucleic Acids Res. 2018, 46, 5239. 29534222 10.1093/nar/gky177 PMC6007322

[119] N. M. Drzeniek , N. Kahwaji , S. Picht , I. M. Dimitriou , S. Schlickeiser , H. Moradian , S. Geissler , M. Schmueck‐Henneresse , M. Gossen , H.‐D. Volk , Adv. Sci. 2024, 11, e2308447. 10.1002/advs.202308447 PMC11151007 38491873

[120] K. Karikó , M. Buckstein , H. Ni , D. Weissman , Immunity 2005, 23, 165. 16111635 10.1016/j.immuni.2005.06.008

[121] K. Karikó , H. Muramatsu , J. Ludwig , D. Weissman , Nucleic Acids Res. 2011, 39, e142. 21890902 10.1093/nar/gkr695 PMC3241667

[122] S. Thakur , A. Sinhari , P. Jain , H. R. Jadhav , Front. Pharmacol. 2022, 13, 1006304. 36339619 10.3389/fphar.2022.1006304 PMC9626821

[123] M. Egli , M. Manoharan , Nucleic Acids Res. 2023, 51, 2529. 36881759 10.1093/nar/gkad067 PMC10085713

[124] L.‐L. Wang , C.‐Q. Wu , Q.‐L. Zhang , Y. Wang , Y. Liu , W.‐J. Yang , S.‐L. Ye , Y. Tian , L. Xu , J. Am. Chem. Soc. 2024, 146, 6665. 38412223 10.1021/jacs.3c12702

